# Introducing carbohydrate patterning in mannose-decorated supramolecular assemblies and hydrogels†

**DOI:** 10.1039/d2cc06064g

**Published:** 2023-01-31

**Authors:** Laura Rijns, Lu Su, Konrad Maxeiner, Giulia Morgese, David Y. W. Ng, Tanja Weil, Patricia Y. W. Dankers

**Affiliations:** a Institute for Complex Molecular Systems (ICMS), Eindhoven University of Technology PO Box 513 Eindhoven 5600 MB The Netherlands p.y.w.dankers@tue.nl; b Department of Biomedical Engineering, Laboratory of Chemical Biology, Eindhoven University of Technology PO Box 513 Eindhoven 5600 MB The Netherlands; c Department of Biomedical Engineering, Laboratory for Cell and Tissue Engineering, Eindhoven University of Technology PO Box 513 Eindhoven 5600 MB The Netherlands; d Leiden Academic Centre for Drug Research (LACDR), Leiden University Einsteinweg 55 Leiden 2333 CC The Netherlands l.su@lacdr.leidenuniv.nl; e Max Planck Institute for Polymer Research Ackermannweg 10 Mainz 55128 Germany; f ZHAW Zurich University of Applied Sciences, School of Engineering, Forschungsbereich Polymere Beschichtungen, Technikumstrasse 9 Winterthur 8400 Switzerland

## Abstract

Benzene-1,3,5-tricarboxamide (BTA) glyco-monomers containing one, two or three mannose units are synthesized and formulated into differently patterned supramolecular glycopolymers through homo-assembly or co-assembly with non-functionalized BTAs. Unfortunately, no cellular activity could be detected. Excitingly, these glyco-BTA monomers could be formulated into hydrogels, paving the way for (immune) cell culture.

## Abstract 2

Mannose patterning is introduced into benzene-1,3,5-tricarboxamide (BTA)-based supramolecular assemblies through copolymerization of fully mannosylated M3 BTA with non-functionalized nBTA, and compared to their asymmetric homopolymer counterparts (M1 and M2 BTA).

## Graphical abstract

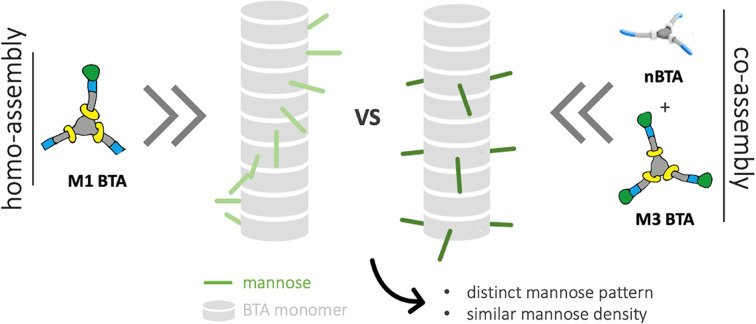

Carbohydrate–protein interactions (CPIs) are abundant in many biological processes and involved in the immune response.^1^ Importantly, many mammalian epithelial cells have a glycocalyx – a coating surrounding the cell surface containing glycoproteins, glycolipids and proteoglycans – which has a crucial role in cell–cell recognition and communication.^2^ Synthetic glycopolymers as mimics of their natural counterparts that present carbohydrates in a multivalent manner are increasingly being designed and synthesized to gain better a understanding of CPIs,^3–9^ of which the mannose – mannose receptor (CD206) is an important one.^10,11^

To illustrate, mannosylated nanoparticles and rods have been created by Besenius, Wurm, De Geest and others, which exhibit specific receptor–ligand interactions to both lectins and dendritic cells (DCs) as compared to poly(ethylene glycol) (PEG) or galactosylated controls,^3,4,12^ and are thus effective and selective in binding biological entities. Feng *et al.* investigated the morphological effects, *i.e.* elongated, rod-like *vs.* spherical, micellar morphology, of glucomannan-decorated particles.^5^ Especially the spherical, micellar morphology could trigger the release of cytokines in macrophages, highlighting the importance of morphology-function relationship of CPIs. Chen *et al.* fabricated glycoparticles bearing galactose and/or mannose, and explored the architectural effects, *i.e.* homogeneously mixed *vs.* blend mixed *vs.* homo-shells, on lectin-binding and macrophage endocytosis performance.^13^ They showed that the homogeneously mixed sugar shells outperform the blend-mixed shells, emphasizing the importance of carbohydrate patterning in CPIs.

Considering the importance of these morphological and architectural effects in effective receptor targeting, supramolecular glyco-assemblies based on non-covalent interactions that exhibit adaptivity in ligand presentation, morphology and dynamicity are emerging as a promising class of glycopolymers.^14,15^ In our lab, we have ample expertise with the benzene-1,3,5-tricarboxamide (BTA) monomer that assembles into different nanostructures, including micelles, sheets and micrometer long fibers, driven by triple amide hydrogen bonding and π–π interactions in the core together with the hydrophobic effect.^16,17^ Our ‘standard’, non-functionalized BTA (nBTA) has three *C*_12_ spacers connected to its core such that water cannot compete for amide hydrogen bonding, and three tetra(ethylene glycol) (EG_4_) arms to provide water solubility (Fig. 1A), forming micrometer long double helical fibers.^18^ In these supramolecular fibers, the spatial rearrangement could easily be adopted through dynamic monomer exchange within the supramolecular polymers to promote binding events.^19^ Besides, control over morphology, supramolecular polymer length and dynamicity could be achieved by tuning the degree of hydrophilicity of the monomers and/or through copolymerization (*i.e.* mixing of different monomers within the same assembly).^7^

**Fig. 1:**
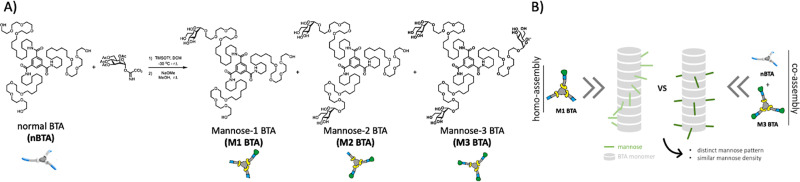
Molecular design and cartoon showing the summary of this study. (A) Molecular design and synthetic route of M*x* BTA (*x* = 1 or 2 or 3) through statistical glycosylation towards nBTA, followed by deprotection with sodium methoxide in MeOH. (B) Illustration showing the copolymerization approach of fully mannosylated M3 BTA with non-functionalized nBTA to afford co-assemblies with similar mannose density, but distinct mannose patterns as compared to their homopolymer analogues.

Recently, the non-covalent synthesis (*i.e.* assembly of monomers into larger supramolecular structures, connected through dynamic, reversible interactions) of a series of saccharide-substituted BTA monomers, including glucose, galactose and mannose, was investigated in detail on the structural and dynamic properties.^7,20,21^ Interestingly, a triple mannose-decorated BTA that forms micelles (M3 BTA) was successfully incorporated into nBTA polymers, resulting in long fibers with increased overall stability, realizing control over the dynamic behavior and morphology.^7^ Surprisingly, the patterning effect of mannose (*i.e.* similar mannose density but different spatial distribution in the fiber) as well as the formation of mannosylated hydrogels and their effect towards (immune) cells remains yet unknown.

Therefore, we here introduce mannose patterning in BTA-based supramolecular assemblies – *via* similar mannose densities but distinct spatial distribution throughout the fiber – and explore their effect on macrophages (Fig. 1B). To achieve this, asymmetric, mono-mannosylated BTA (M1 BTA) and double-mannosylated BTA (M2 BTA) homo-assemblies were compared to their corresponding copolymers consisting of fully mannosylated BTA (M3 BTA) and nBTA (Copolymer_1 with nBTA : M3 BTA = 2 : 1 (mol) and Copolymer_2 with nBTA : M3 BTA = 1 : 2) (Fig. 1A). The non-covalent synthesis of the homo-assemblies and their corresponding co-assemblies are investigated in MQ-H_2_O. Furthermore, mannosylated supramolecular gels are fabricated by increasing the concentration above their critical gelation concentration (CGC).

The asymmetric M1 and M2 BTA molecules were synthesized by direct glycosylation towards the nBTA with active mannose imidate as the donor and trimethylsilyl trifluoromethanesulfonate as the promoter, followed by deprotection with sodium methoxide, to obtain pure (purity >99%) M1 and M2 BTA with an overall yield of 14% (305 mg) and 26% (629 mg), respectively (Fig. 1A). The successful synthesis of M1 and M2 BTA was confirmed by a combination of nuclear magnetic resonance (NMR) measurements (^1^H, ^13^C and 2D NMR), Fourier-transform infrared (FT-IR) spectroscopy and with matrix assisted laser absorption/ionization-time of flight (MALDI-TOF) mass spectrometry (Fig. S1–S6, ESI†).

nBTA^17^ and M3 BTA^7^ have shown to form micrometer-long fibers and micelles, respectively. Both homo-assembled M1 and M2 BTA show maximum absorptions in UV at 211 and 227 nm, which is also observed for their counterparts Copolymer_1 and _2 (Fig. 2A). This almost completely overlaps with the absorption spectrum of nBTA, indicative for fibers (Fig. 2A). However, the absorption peaks for M2 BTA and its Copolymer_2 are less pronounced than that of nBTA, which might indicate different fibrous structures. Nile red assays indicated a significant blue shift for the homo-assembled BTAs as well as for the co-assemblies (610 nm), when compared with the micelle-forming M3 BTA (630 nm), demonstrating the presence of a hydrophobic pocket (Fig. 2B). Static light scattering (SLS) showed a dependence of the Rayleigh ratio on the scattering angle for all homo- and co-assembled BTAs, except for the micelle-forming M3 BTA, indicating elongated structures (Fig. 2C). Cryo-TEM further confirmed the morphology of micrometer long nanofibers with a diameter of 6 ± 2 nm for M1 and M2 BTA as well as for Copolymer_1 and _2 (Fig. 2D – representative image, Fig. S7–S10, ESI†). Micro Differential Scanning Calorimetry (microDSC) suggested a co-assembled state for Copolymer_1 and Copolymer_2 rather than self-sorting, as suggested by the absence of nBTA endothermic peaks in the curves (Fig. 2E and Fig. S13, ESI†). M1 BTA showed an endothermic peak in microDSC at higher temperature (80 °C) than Copolymer_1 (75 °C), suggesting different microstructure and higher thermal stability for M1 BTA (Fig. 2E). On the contrary, M2 BTA showed an endothermic peak at 58 °C and Copolymer_2 at 66 °C, suggesting less thermal stability for M2 BTA (Fig. 2E). Noteworthy, for M1 BTA, M2 BTA and both Copolymer_1 and _2, the endothermic peaks in microDSC appear at higher temperatures in all cases than the transition in the variable temperature (vt) UV heating curves. To illustrate, the transition in microDSC is at 80 and 75 °C for M1 BTA and Copolymer_1 (Fig. 2E), respectively, *vs.* at 65 °C for both in vt UV (Fig. S11, ESI†). Likewise for M2 BTA and Copolymer_2, the transition in microDSC occurs respectively at 58 and 66 °C (Fig. 2E) *vs.* at 45 and 60 °C for vt UV (Fig. S12, ESI†). These differences might arise from the different heating rate and concentrations used for the different techniques. Interestingly, the CD spectra of M1 BTA (Fig. S14, ESI†) and of M2 BTA (Fig. 2F) showed significant ageing effect. Although the exact molecular mechanism for the increased CD signal after ageing remains questionable, it is proposed that ageing is required for the polymer to reach its maximal preferred helical bias, accompanied by decreased dynamics.^22^ Besides, M2 BTA showed a more profound CD signal (Fig. 2F; peaks at 210 and 250 nm) as compared to its Copolymer_2. This indicates that the mannose pattern within the fibers should be different. This is of great interest for further cell experiments. In conclusion, micelle-forming M3 BTA was successfully co-assembled with nBTA to form supramolecular copolymers with similar mannose density but different mannose pattern as their corresponding homo-assemblies (M1 and M2 BTA).

**Fig. 2:**
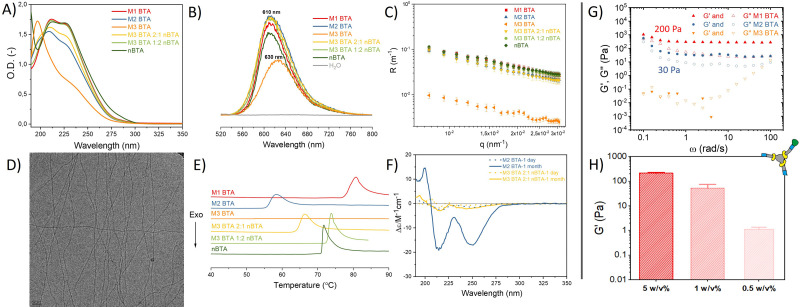
Characterization of the supramolecular mannose BTA homo-assemblies and their corresponding co-assemblies as well as mechanical characterization of mannosylated supramolecular hydrogels. (A) UV spectra (50 μM), (B) Nile red assay (50 μM), (C) SLS profile (500 μM), (D) representative Cryo-TEM image of M1 and M2 BTA (500 μM). Scale bar = 50 nm. (E) microDSC profiles (500 μM), and (F) CD spectra (50 μM) of BTAs in MQ-H_2_O. (G, H) Mechanical characterization of supramolecular mannosylated BTA hydrogels using rheology, with in (*G*) angular frequency sweep plot at 1% strain showing the storage (*G*′) and loss modulus (*G*′′) at 5 w/v% and in (H) *G*′ quantified at different concentrations of M1 BTA to determine its CGC.

Next, hydrogels were fabricated with the mannosylated BTAs. The CGC of M1 BTA was found to be ∼0.5 w/v%. M1 BTA could still withstand its own weight at 0.5 and 1 w/v% concentrations in vial-inversion tests (Fig. S16A, ESI†), but forms very soft gels with a *G*′ of only a few Pa and of ∼50 Pa for the 0.5 and 1 w/v% samples, respectively (Fig. 2H and Fig. S17, ESI†). On the contrary, M2 BTA could not pass the vial-inversion test at 0.5 and 1 w/v% concentrations (Fig. S16B, ESI†) and appeared as a liquid. This indicates that its CGC is higher than 1 w/v%, but lower than 5 w/v%. Rheology performed at 5 w/v% concentrations revealed that M1 and M2 BTA form soft gels with a *G*′ ∼200 Pa and 30 Pa, respectively (Fig. 2G and Fig. S15, ESI†). However, M3 BTA remained a liquid with *G*′′ exceeding *G*′ and containing higher tan(*δ*) values than M1 and M2 BTA, indicating a higher viscous-like behavior for M3 BTA (Fig. 2G and Fig. S15, ESI†). The difference in bulk stiffness between M1 and M2 BTA could be explained by the longer fibrous structures of M1 BTA in comparison with M2 BTA, resulting in a stronger gel. We hypothesize that the fibers of both M1 and M2 BTA entangle at these concentrations also due to carbohydrate–carbohydrate interactions, resulting in the formation of a gel. Whereas the more hydrophilic M3 BTA that forms micelles in diluted state prevents the formation of a gel. It should also be realized that fewer molecules of M3 BTA (*c* = 28.2 mM) are present in the 5 w/v% samples as compared to M1 (*c* = 34.5 mM) and M2 BTA (*c* = 31 mM), owing to its higher molecular weight. Therefore, a control sample of M3 BTA at 5 w/v% was created, containing a similar molar concentration (*c* = 34.5 mM) to that of M1 BTA at 5 w/v%. The control remained a liquid that could not hold its own weight during a vial-inversion test (Fig. S16C, ESI†). Importantly, the mannosylated gels exhibit self-healing behavior as demonstrated with the M1 BTA gel (Fig. S17B, ESI†).

Next, the biological effect of the mannose-decorated homo- and co-assemblies on immune cells was tested. Therefore, the fiber stability in complex media is of great importance. In absence and presence of fetal bovine serum (FBS), an abundant component in cell medium, the presence of fibers, but also aggregated structures, was confirmed using total internal reflection microscopy (TIRF) (Fig. 3A). This indicates that serum does not affect the structural properties homo-assemblies nor the co-assemblies. None of the mannose BTAs affect the cell viability of RAW 264.7 macrophages, important cells of the immune system, up to 100 μM concentrations (Fig. 3B and Fig. S18, ESI†). For efficient targeting of the immune cells using mannose as ligand, the expression of CD206 on the cell surface is crucial. Flow cytometry experiments confirmed the presence of the CD206 (∼50%) on the macrophages (Fig. S19, ESI†). Subsequent internalization experiments showed endocytosis for all assemblies by the macrophages already after incubation of 2 h (Fig. 3C and Fig. S20, ESI†), agreeing with other studies that also reveal endocytosis of glycoparticles by immune cells.^3,23^ However, internalization was also observed for nBTA, suggesting that the endocytosis is not CD206-mediated, in contrast to other studies.^3,13^ The internalization could be caused by the origin of the cells, as macrophages are known for their phagocytic capabilities, but could also be Cy5-mediated, as Cy5 is known to facilitate cellular uptake caused by its tendency to bind the mitochondria.^24,25^ To study whether metabolic pathways, *i.e*. oxidative phosphorylation or glycolysis, are affected during and after endocytosis of the BTA assemblies, extracellular flux assays were executed to reveal how the integrity of intracellular structures are influenced, thereby affecting cell viability. Minimal increase in oxygen consumption rate (indicative for oxidate phosphorylation) or extracellular acidification rate (indicative for glycolysis) was detected with 10 h incubation (Fig. S21 and S22, ESI†), indicating limited energy consumption by the macrophages during the internalization and that associated binding events by the BTAs do not increase the metabolic load of the cells.

**Fig. 3:**
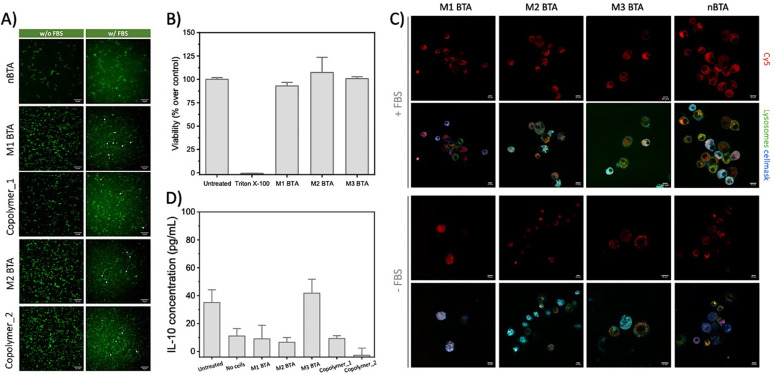
Fiber stability, toxicity and cell internalization experiments. (A) Representative TIRF images of the homo- and co-assemblies (BTA = 2.5 μM, with 5% of BTA-Cy3) with and without the presence of FBS at 20 °C, showing that the fibrous morphology could be maintained in cell-like environments. Scale bar = 10 μm. (B) Cell viability assay of 100 μM BTA assemblies towards RAW264.7 macrophages as determined with a resazurin assay. The cell viability is normalized on cells that were maintained in untreated medium (*i.e.* medium control). (C) Internalization experiment in which RAW 264.7 macrophages were incubated with Cy5-labeled BTAs (*c* = 25 μM total, with 5% BTA-Cy5^26^) in presence and absence of FBS for 2 h, and stained thereafter. Scale bar = 10 μm. (D) ELISA assay to quantify the release of IL-10 in RAW 264.7 macrophages after treatment with 25 μM mannose BTAs for 24 h.

To explore if the mannosylated glycopolymers could activate CD206 in macrophages, their IL-10 cytokine release was quantified using Enzyme-linked Immuno Sorbent Assay (ELISA) assays. Unfortunately, no IL-10 release could be detected after 24 h incubation (Fig. 3D and Fig. S23, ESI†). Lipopolysaccharide (LPS) was used as positive control and showed extremely high IL-10 release of up to 6000 pg mL^−1^ IL-10 for the 10 μg mL^−1^ condition, while mannan (*i.e.* poly mannose) could not induce any release (Fig. S24, ESI†). Although similar concentrations of BTA or LPS were used (100 μM BTA roughly equals 100–200 μg mL^−1^ BTA, which is in the same order of magnitude as used for LPS; 0.01–100 μg mL^−1^ and mannan; 100 μg mL^−1^), it should be realized that LPS is a more complex mix of various carbohydrates. Also, our BTA assemblies could interact with bovine serum albumin (BSA)^20^ that is present in FBS in complex media. A possible scenario might include the complete ‘blocking’ of the mannose residues on the BTA assemblies by BSA. Another explanation could be the dynamicity of the supramolecular assemblies. In case the monomer exchange rate is faster than the timeframe in which CD206 could recognize and bind mannose, binding between the receptor and mannose-decorated fibers will be limited.

To conclude, mannose patterning was introduced in BTA-based, supramolecular assemblies through supramolecular copolymerization of fully mannosylated BTA (M3 BTA) and nonfunctionalized BTA (nBTA) to produce a series of BTA copolymers that were compared with their asymmetric, homo-assembled counterparts (M1 BTA and M2 BTA). Unfortunately, no biological effect could be detected. Interestingly, mannosylated hydrogels could be formed for M1 BTA (CMC ∼0.5 w/v%) and M2 BTA (1 w/v% < CMC < 5 w/v%), whereas the more hydrophilic M3 BTA remained a liquid. We anticipate that these mannose-patterned supramolecular glycopolymers and hydrogels exhibiting inherent dynamicity and great tunability allow us to arrive at deeper understanding of multivalent CPIs, which are essential to many biological processes.

We thank Svenja Ehrmann and Sandra Schoenmakers for measuring the cryoTEM images. We thank prof. Bruno de Geest and prof. Bert Meijer for useful discussions. We acknowledge financial support from NWO (TOP-PUNT Grant 10018944), the Dutch Ministry of Education, Culture and Science (Gravitation programs 024.001.035 and 024.003.013), the European Commission (SYNMAT- 788618-1).

## Conflicts of interest

There are no conflicts to declare.

## Supplementary Material

CC-059-D2CC06064G-s001
