# Supplementary material for: Introducing carbohydrate patterning in mannose-decorated supramolecular assemblies and hydrogels
Source: Chem Commun (Camb). 2023 Jan 31;59(15):2090–3. doi: 10.1039/d2cc06064g (PMC9933453; doi:10.1039/d2cc06064g)
Supplement: CC-059-D2CC06064G-s001 [file CC-059-D2CC06064G-s001.pdf]

Electronic Supplementary Material (ESI) for ChemComm.
This journal is © The Royal Society of Chemistry 2023

Supplementary information for

Introducing carbohydrate patterning in mannose-decorated supramolecular assemblies and hydrogels

Laura Rijns[a,b,c], Lu Su[a,d†], Konrad Maxeiner[e], Giulia Morgese[f], David Y. W. Ng[e], Tanja Weil[e], Patricia Y. W. Dankers[a,b,c†]

† Corresponding author

[a] *Institute for Complex Molecular Systems (ICMS), Eindhoven University of Technology, PO Box 513, 5600 MB Eindhoven, The Netherlands*
[b] *Department of Biomedical Engineering, Laboratory of Chemical Biology, Eindhoven University of Technology, PO Box 513, 5600 MB Eindhoven, The Netherlands*
[c] *Department of Biomedical Engineering, Laboratory for Cell and Tissue Engineering, Eindhoven University of Technology, PO Box 513, 5600 MB Eindhoven, The Netherlands*
[d] *Leiden Academic Centre for Drug Research (LACDR), Leiden University, Einsteinweg 55, Leiden 2333 CC, The Netherlands.*
[e] *Max Planck Institute for Polymer Research, Ackermannweg 10, 55128 Mainz, Germany.*
[f] *ZHAW Zurich University of Applied Sciences, School of Engineering, Forschungsbereich Polymere Beschichtungen, Technikumstrasse 9, 8400 Winterthur, Switzerland*

Materials and Methods

Materials: All reagents and chemicals were obtained from commercial sources at the highest purity available and used without further purification unless stated otherwise. All solvents were of AR quality and purchased from Biosolve. Water was purified on an EMD Millipore Milli-Q Integral Water Purification System. Reactions were followed by thin-layer chromatography (precoated 0.25 mm, 60-F254 silica gel plates from Merck), and flash chromatography was run with silica gel (40–63 µm, 60 Å from Screening Devices b.v.). Dry solvents were obtained with an MBRAUN Solvent Purification System (MB-SPS). Automated column chromatography was performed using Biotage® SNAP-KP SIL cartridges.

Nuclear Magnetic Resonance (NMR): NMR spectra were recorded on Bruker 400 MHz Ultrashield spectrometer (400 MHz for ^{1}H NMR; 100 MHz for ^{13}C NMR). Deuterated solvents used are indicated in each case. Chemical shifts (δ) are expressed in ppm and are referred to the residual peak of the solvent peak. Multiplicity is abbreviated as s: singlet; d: doublet; t: triplet; dd: doublet of doublets m: multiplet.

Matrix assisted laser absorption/ionization-time of flight (MALDI-TOF) mass spectra: MALDI-TOF were obtained on a PerSeptive Biosystems Voyager DE-PRO spectrometer using α-cyano-4-hydroxycinnamic acid (CHCA) or trans-2-[3-(4-*tert*-butylphenyl)-2-methyl-2-propenylidene]-malononitrile (DCTB) as matrix.

Liquid chromatography mass spectrometry (LC–MS): LC-MS was performed on a system consisting of the following components: a Shimadzu SCL-10A VP system controller with Shimadzu LC-10AD VP liquid chromatography pumps with an Alltima C18 3 u (50 × 2.1 mm) reversed-phase column and gradients of water–acetonitrile supplemented with 0.1% formic acid, a Shimadzu DGU 20A3 prominence degasser, a Thermo Finnigan surveyor auto sampler, a Thermo Finnigan surveyor PDA detector, and a Thermo Scientific LCQ Fleet. Gradients were run from 5% MeCN to 100% MeCN over a 15 min period.

Fourier-transform infrared (FT-IR) spectroscopy: FT-IR spectra were measured on a Perkin Elmer Spectrum Two FT-IR spectrometer, equipped with a Perkin Elmer Universal ATR Sampler Accessory. The solid state spectra were recorded at room temperature from 400 cm^{-1} to 4000 cm^{-1} and averaged over 16 scans.

Thin layer chromatography (TLC): TLC was executed using 60-F254 silica gel plates (Merck). Compounds were visualized with an ultraviolet lamp (254 nm).

Static Light Scattering (SLS): Measurements were performed on an ALV Compact Goniometer (CGS-3) Multi-Detector (MD-4) equipped with an ALV-7004 Digital Multiple Tau Real Time Correlator and an Nd-YAG laser operating at a wavelength of 532 nm. The scattering intensity was detected over the angular range of 30−150° with steps of 5° or 10 °, with 10 runs of 10 s per angle. Samples were prepared at a 250 µM or 0.8 mM concentration of **nBTA** in MQ water, and measured in light scattering tubes with an outer diameter of 1 cm. As a reference, samples of only the corresponding solvent and only toluene were measured. Water was filtered with a 0.2 µm syringe filter (Supor membrane, PALL Corporation), and toluene was filtered with a 0.2 µm syringe filter (PTFE membrane, Whatman). The measurements were analyzed with AfterALV (1.0d, Dullware) to remove measurements showing obvious scattering from dust. The Rayleigh ratio as a function of the angle was computed using the equation below with toluene as a reference

$$R(\theta) = \frac{I_{sample(\theta)} - I_{solvent(\theta)}}{I_{toluene(\theta)}} \times R_{toluene(\theta)} \times \frac{{n_{solvent}}^2}{{n_{toluene}}^2} \qquad (1)$$

with I_{sample} the count rate of the sample solution, I_{solvent} the count rate for the solvent (water), and I_{toluene} the count rate for toluene. R_{toluene} is the known Rayleigh ratio of toluene (2.1 × 10^{-2} m^{-1} at 532 nm), n_{solvent} is the refractive index of the solvent (1.333 for water), and n_{toluene} is the refractive index of toluene (1.497)

Detection angles were converted to scattering vectors (q) according to

$$q = (4\pi n_0 / \lambda) \times sin\ (\theta / 2) \qquad (2)$$

Ultraviolet-visible (UV-vis) absorbance spectra: UV-vis spectra were recorded on and a Jasco V-650 UV-vis spectrometer or a Jasco V-750 UV-vis spectrometer with a Jasco ETCT-762 temperature controller.

Transmission electron cryo-microscopy: Vitrified films were prepared in a 'Vitrobot' instrument (FEI Vitrobot™ Mark IV, FEI Company) at 22 °C and at a relative humidity of 100%. In the preparation chamber of the 'Vitrobot' samples were applied on grids, which were surface plasma treated just prior to use (Cressington 208 carbon coater operating at 5 mA for 40 s). Excess sample was removed by blotting using filter paper with a blotting force of -1, and the thin film thus formed was plunged (acceleration about 3 g) into liquid ethane just above its freezing point. Liquid samples were vitrified on Quantifoil grids (R 2/2, Quantifoil Micro Tools GmbH) by applying 3 µL of the sample and blotting away the sample for 3 s. Samples with a high viscosity were vitrified within 15 min after preparing the samples. Vitrified films were prepared

by applying 2 µL of the sample on Lacey grids (LC200-Cu, Electron Microscopy Sciences) and blotting away the sample for 5 s. Due to the high viscosity of the sample not all material could be blotted away, resulting in a thick layer of vitrified water and only 10-20% of the grids could be imaged for those samples. Vitrified films were transferred into the vacuum of a CryoTITAN equipped with a field emission gun that was operated at 300 kV, a post-column Gatan energy filter with a slit width of 20 eV, and a 2048 x 2048 Gatan CCD camera. Vitrified films were observed in the CryoTITAN microscope at temperatures below -170 °C. Micrographs were taken at low dose conditions, starting at a magnification of 6500 with a defocus setting of -40 µm, and at a magnification of 24000 with a defocus setting of -10 µm or -5 µm. The contrast was increased with 20% for all images.

Micro-differential scanning calorimetry (Micro-DSC): Micro-DSC was performed on a TA Instruments Multicell DSC. About 1.0 mL of material was prepared in Hastelloy ampoules and characterized using the following heating program: Equilibrate at 25 °C, 25 °C to 90 °C at a rate of 60 °C/h, equilibrate for 15 min, 90 °C to 5 °C at 60 °C/h, equilibrate for 15 min, 5 °C to 90 °C at 60 °C/h, equilibrate for 15 min, 90 °C to 5 °C at 60 °C/h, equilibrate for 15 min, 5 °C to 90 °C at 60 °C/h, equilibrate for 15 min, 90 °C to 25 °C at 60 °C/h. Baseline curves of MQ water were subtracted from sample curves.

Total internal reflection fluorescence (TIRF) microscopy: TIRF images were acquired with a Nikon N-STORM microscopy system. Sample was excited using 561 nm laser. Fluorescence was collected using a Nikon×100, 1.4NA oil immersion objective and passed through a quad-band pass dichroic filter (97335 Nikon). Images were recorded with an EMCCD camera (ixon3, Andor, pixel size 0.17 µm). The samples were imaged in a µ-Slide 8 Well plate with No. 1.5 coverslip bottom suitable for microscopy.

Cell culture: RAW264.7 macrophages were cultured in DMEM:F12 1:1 medium containing L-Glu and HEPES (ATCC, 30-2006) supplemented with 1 v/v% penicillin/streptomycin (P/S) and 10 v/v% fetal bovine serum (FBS) at 37 °C and 5% CO_2. Medium was changed every 5 days and cells were passaged at 95% confluency using trypsin/EDTA. Cells were cultured from the 12th passage until passage number 31.

Cell viability - resazurin assay: Resazurin assays were performed to assess whether the molecules showed toxic effects to the cells. To this end, RAW264.7 macrophages were seeded at a density of 5×10^4 cells/well in a 96-well plate, respectively, and were maintained under standard culturing conditions until cells were grown to 60-70% confluence. Next, the cells were grown in the presence of the UV-sterilized mannose BTAs in 100 µL fresh culture medium for an additional 24 h. To assess cell viability, 44 µM resazurin in

fresh culture medium was added to the cells. The cells were incubated under standard culturing conditions for 3 h. Viable cells convert non-fluorescent resazurin into fluorescent resorufin (λ_{ex} = 530 nm, λ_{em} = 590 nm), which was measured on a Synergy HT plate reader in 90 µL in triplicate per well. Each condition was tested 3 times. Background subtraction (i.e. wells containing only medium, without cells) was carried out for all measurements. Cell viability in percentage is presented relative to the viability of RAW264.7 macrophages that were cultured in untreated medium, which was set at 100% cell viability. Cells exposed to 0.5 v/v% Triton X-100 in phosphate buffered saline (PBS) were used as negative control.

Quantification of CD206 expression on cell membrane - flow cytometry: Cells were trypsinized, centrifuged at 300 rpm for 5 min and resuspended in PBS with 2 v/v% bovine serum albumin (BSA) to reduce non-specific binding. For the RAW264.7 macrophages (3.33×10^5 cells per sample), the cells were first blocked with 5 v/v% BSA in PBS to reduce non-specific binding. The sample of interest was incubated with an anti-mouse allophycocyanin (APC) primary anti-CD206 antibody (2 µg/mL) for 30 min at room temperature in PBS containing 2 v/v% BSA, while the isotype control sample was incubated with a rat, APC isotype control antibody (not specific for CD206) at similar concentrations to correct for non-specific binding of the antibody. The samples were centrifuged at 300 rpm for 5 min after each antibody incubation step and washed with PBS. Unstained cells were used to correct for background and autofluorescence of the cells. All antibodies were purchased from Biolegend. The obtained data was analyzed using Flow Jo software (Tree Star, Ashland, OR, USA).

Quantification of IL-10 cytokine release – ELISA: ELISA assays. To this end, RAW264.7 macrophages were seeded at a density of 5×10^4 cells/well in a 96-well plate. Simultaneously with cell seeding, UV-sterilized mannose BTAs, poly-mannose or LPS were added in a total of 100 µL culture medium, and the cells were maintained under standard culturing conditions for 24 h. After 24 h, 90 µL of the cell medium was collected and quantified for IL-10 using ELISA (Biolegend, IL-10 mouse Max Deluxe) according to the manufacturer's protocol. If necessary, the samples containing cell medium were stored at -80 °C until the ELISA experiment was performed. A standard curve was measured ranging from 0 – 2000 pg/mL IL-10 and the data was fitted using a 2nd degree polynomial fit. Non-stimulated, untreated cells were used as negative control and cells stimulated with LPS as positive control.[1]

Internalization studies – fluorescent confocal microscopy: RAW 264.7 macrophages were seeded in Ibidi µ-angiogenesis slides prior to the experiment and allowed to adhere. Stock solutions of the mannose BTA

assemblies containing **BTA-Cy5**[2] (5%) for visualization were prepared at 250 µM stock concentration and allowed to incubate overnight in the dark. The next day, the assemblies were added to the cells (5 µL with 45 µL medium) to obtain a final concentration of 25 µM BTAs. The cells with the BTA assemblies were incubated for 2 h at 37 °C with 5 % CO_2. Next, the samples were washed with PBS, followed by fixation for 10 min with 3.7 v/v% formaldehyde and washing with PBS twice afterwards. Then, the cells were stained with a lysosomal (green) and cell (blue) mask for 1 h from Invitrogen according to the manufacturer's procedure. Finally, the samples were washed with PBS. Immediately thereafter, the samples were imaged on a Leica TCS SP5 inverted confocal microscope using 10× (HCX PL APO CS 10.0 × 0.4 DRY UV), 20× (HCX PL APO CS 20.0 × 0.7 DRY UV) and 40× (HCX PL APO CS 40.0 × 1.1 water UV) objectives.

Metabolic changes - seahorse Metabolism Flux Assay: Metabolic changes of RAW 264.7 macrophages during the incubation with the BTA compounds were monitored using the Seahorse XFe96 Analyzer (Agilent), measuring Extracellular Acidification Rate (ECAR) and Oyxgen Consumption Rate (OCR). RAW 264.7 macrophages (6×10^5 cells/well) were seeded in XFe96 plates using standard DMEM the day before the assay and incubated at 37 °C, 5% CO_2. The cartridge for sample loading was incubated with 200 µL calibration solution at 37°C in a non-CO_2 incubator overnight. On the day of the assay cells were washed with XF DMEM supplemented with glucose (10 mM), glutamine (2 mM) and pyruvate (1 mM). 180 µL XF DMEM was added in each well and the microplate was placed at 37 °C in a non-CO_2 incubator for 45 min. 20 µL samples at a 10-fold concentration diluted in MQ H_2O were loaded in Port A of the cartridge. Metabolism was monitored measuring ECAR and OCR after addition of BTA compounds. First experiments were performed measuring the changes of ECAR and OCR over a total period of 10 h after the addition of BTA compounds. In addition, Seahorse XF Cell Mito Stress Assay was used to investigate the mitochondrial function of BTA treated RAW 264.7 macrophages. After 4 h incubation with the BTA compounds, oligomycin (Port B, 2.5 µM), carbonylcyanide-4 (trifluormethoxy) fenylhydrazon, an uncoupler of mitochondrial oxidative phosphorylation (Port C, 1.0 µM), and a mixture of rotenone and antimycin A (Port D, 0.5 µM) were added to the cells in 30 min intervals.

Rheology: Rheological measurements of mannose BTA samples were carried out on a discovery hybrid rheometer (DHR-3, TA Instruments). Hydrogels were prepared according to the sample preparation method and scooped when possible or else pipetted (**M3 BTA**) onto the rheometer. Hydrogels were analyzed using a cone plate geometry (diameter=25 mm) at a gap height of 49 µm. All measurements were performed in duplo (except for the **M1 BTA** concentration series) and measured at room temperature. A solvent trap was used to minimize sample drying during the measurements. Strain sweep

experiments were measured at strains ranging from 1 to 100%, at a constant frequency of 1 rad/s. Frequency sweeps were performed at frequencies ranging from 100 to 0.1 rad/s, at a constant strain of 1%. The self-healing experiment was performed 1 h after gel preparation. The gel was subjected to the following cycle: 1% strain at 1 rad/s for 50 s, 1000% strain at 1 rad/s for 50 s to achieve gel rupture; 1% strain at 1 rad/s until the gel was self-healed to its initial stiffness.

Synthesis procedures

a. The compounds 2,3,4,6-tetra-*O*-acetyl-α-D-mannopyranosyl trichloroacetimidate[3] and BTA-OEG$_4$[4] *N*^{1}, *N*^{3}, *N*^{5}-Tris(dodecyl-tetra(ethylene glycol)-*O*-α-D-mannopyranoside)benzene-1,3,5-tricarboxamide (**M3 BTA**)[5] were synthesized according to literature procedures.

b. The synthesis of asymmetric BTAs, *N*^{1}-(dodecyl-tetra(ethylene glycol)-*O*-α-D-mannopyranoside)- *N*^{3}*,* *N*^{5}-bis(dodecyl-tetra(ethylene glycol)-benzene-1,3,5-tricarboxamide (**M1 BTA**) and *N*^{1}, *N*^{3}-bis(dodecyl-tetra(ethylene glycol)-*O*-α-D-mannopyranoside)-*N*^{5}-(dodecyl-tetra(ethylene glycol)-benzene-1,3,5-tricarboxamide (**M2 BTA**).

An oven-dried round bottom flask containing a stir bar and 4 Å molecular sieves was charged with 2,3,4,6-tetra-*O*-acetyl-α-D-mannopyranosyl trichloroacetimidate (1.1 g, 2.3 mmol), **nBTA** (1.9 g, 1.5 mmol) and dry dichloromethane (DCM, 10 mL). The mixture was stirred at -30 °C under an Ar atmosphere for 20 min. To the mixture, trimethylsilyl trifluoromethanesulfonate (TMSOTf, 0.27 mL, 1.5 mmol) in DCM (2 mL) was added dropwise over 5 min. Progress was monitored by thin-layer chromatography (TLC) (DCM/MeOH = 49:1) and the reaction was quenched with trimethylamine (0.2 mL) after 30 min. After the mixture was first warmed to room temperature and then concentrated, the crude material was used for next step without further purification.

To a solution of BTA-EG$_4$-Man*O*Ac dissolved in dry DCM (2 mL) and methanol (MeOH, 8 mL) under Ar at room temperature, sodium methoxide (1.0 g, 18.4 mmol, 2 *eq*. to *O*Ac group) was added while stirring. Progress was monitored by TLC and the reaction was quenched with Dowex® 50WX8 hydrogen form resin after 2 h. The resin was removed by filtration and the filtrate was concentrated under reduced pressure. The crude product was purified by reversed-phase chromatography (H_2O/MeOH gradient 50/50–20/90 v/v) to give **M1 BTA** and **M2 BTA** as white, fluffy solids after lyophilization (304.5 mg, 14% of **M1 BTA**, and 628.7 mg, 26% of **M2 BTA** in two steps, respectively). Noteworthy, due to the resolution limitation, the signals of BTA arms with (*C$_{12}$-EG$_4$*-Man) and without (*C$_{12}$-EG$_4$*) mannose substitution are almost identical in NMR spectra.

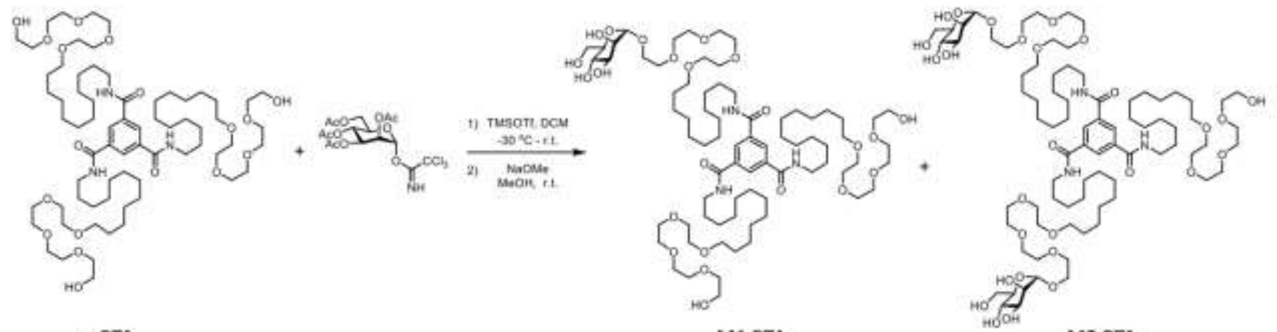

nBTA M1 BTA M2 BTA

M1 BTA

^{1}H NMR (400 MHz, MeOD) δ 8.37 (s, 3H), 4.79 (d, *J* = 1.7 Hz, 1H), 3.85 – 3.80 (m, 3H), 3.72 (d, *J* = 5.5 Hz, 1H), 3.69 – 3.60 (m, 39H), 3.56 (td, *J* = 5.7, 3.8 Hz, 12H), 3.43 (dt, *J* = 23.6, 6.9 Hz, 12H), 1.60 (dp, *J* = 33.9, 6.9 Hz, 12H), 1.45 – 1.25 (m, 49H).

^{13}C NMR (100 MHz, MeOD) δ 168.60, 136.86, 129.75, 101.76, 74.61, 73.67, 72.56, 72.38, 72.12, 71.62, 71.60, 71.58, 71.55, 71.54, 71.40, 71.15, 68.63, 67.77, 62.96, 62.22, 49.71, 41.22, 30.73, 30.70, 30.68, 30.58, 30.46, 28.09, 27.21.

MALDI-TOF-MS: Calculated for $C_{75}H_{139}O_{23}N_3$: 1449.98 g/mol; found $[M+Na]^+$: 1472.97 g/mol.

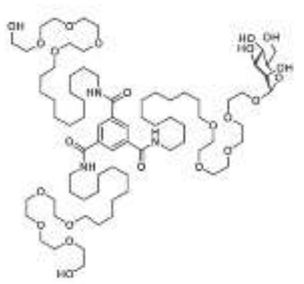

M1 BTA

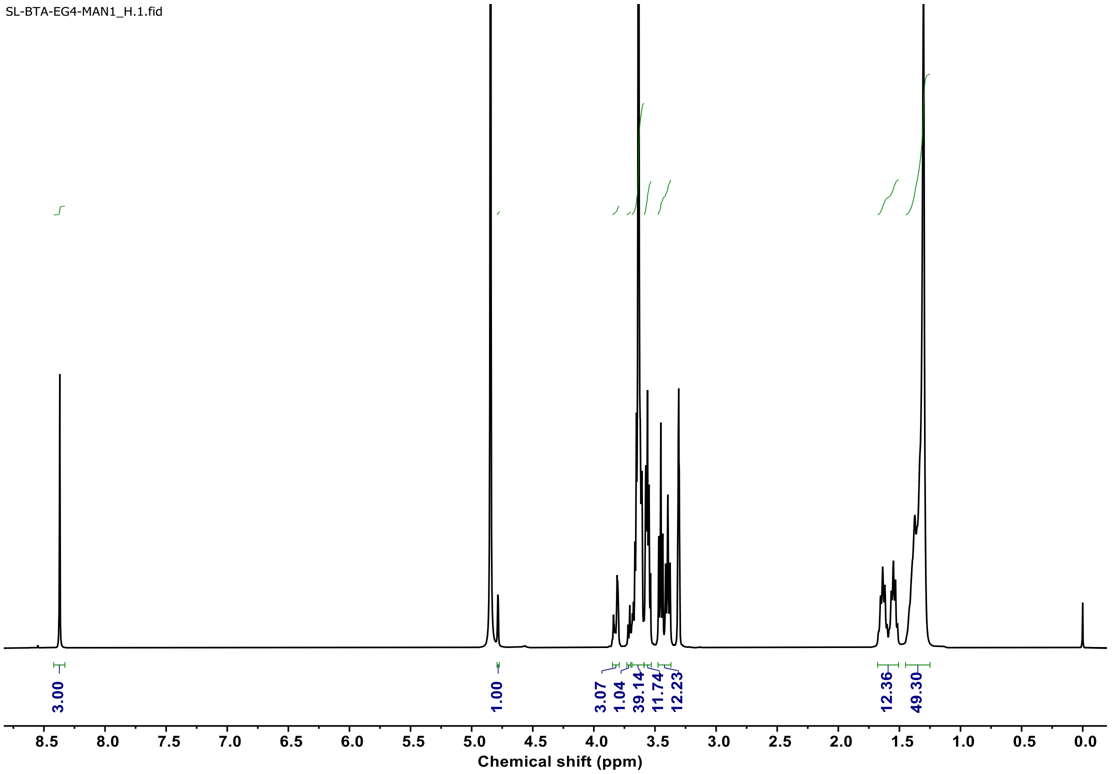

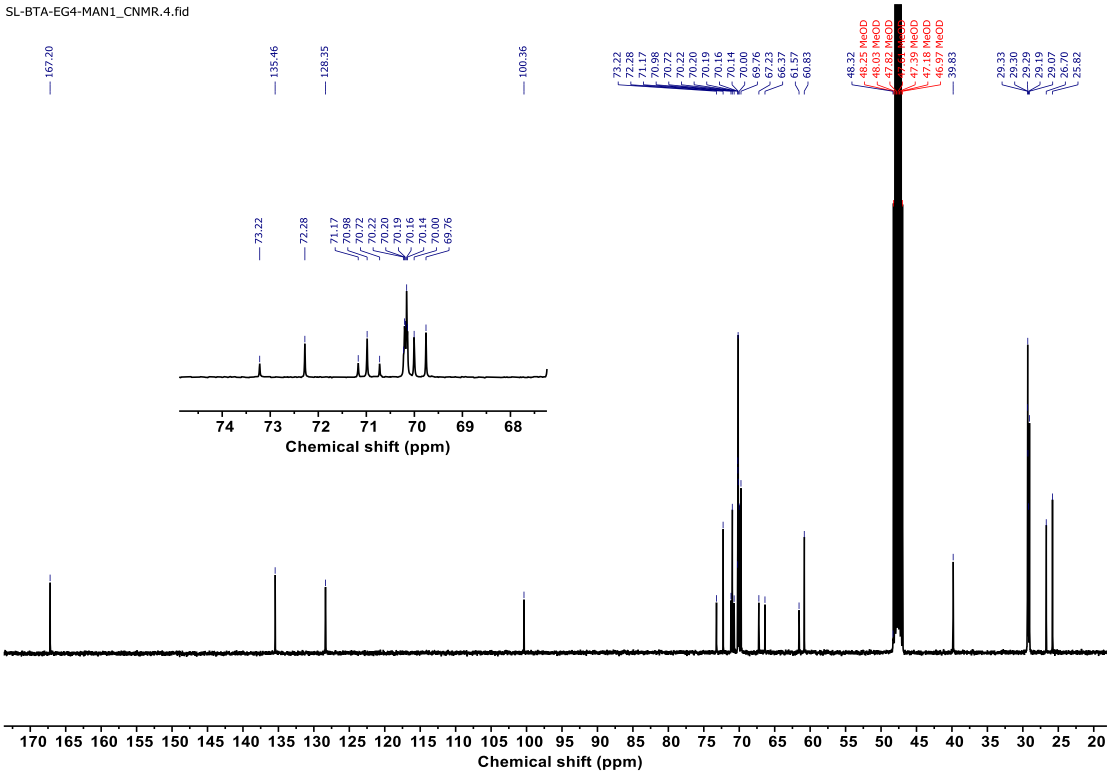
SL-BTA-EG4-MAN1_CNMR.4.fid
167.20
135.46
128.35
100.36
73.22
72.28
71.17
70.98
70.72
70.72
70.22
70.20
70.19
70.16
70.14
70.00
69.76
67.23
66.37
61.57
60.83
48.32
48.25 MeOD
48.03 MeOD
47.82 MeOD
47.39 MeOD
47.18 MeOD
46.97 MeOD
39.83
29.33
29.30
29.29
29.19
29.07
26.70
25.82
73.22
72.28
71.17
70.98
70.72
70.22
70.20
70.19
70.16
70.14
70.00
69.76
74 73 72 71 70 69 68
Chemical shift (ppm)
170 165 160 155 150 145 140 135 130 125 120 115 110 105 100 95 90 85 80 75 70 65 60 55 50 45 40 35 30 25 20
Chemical shift (ppm)

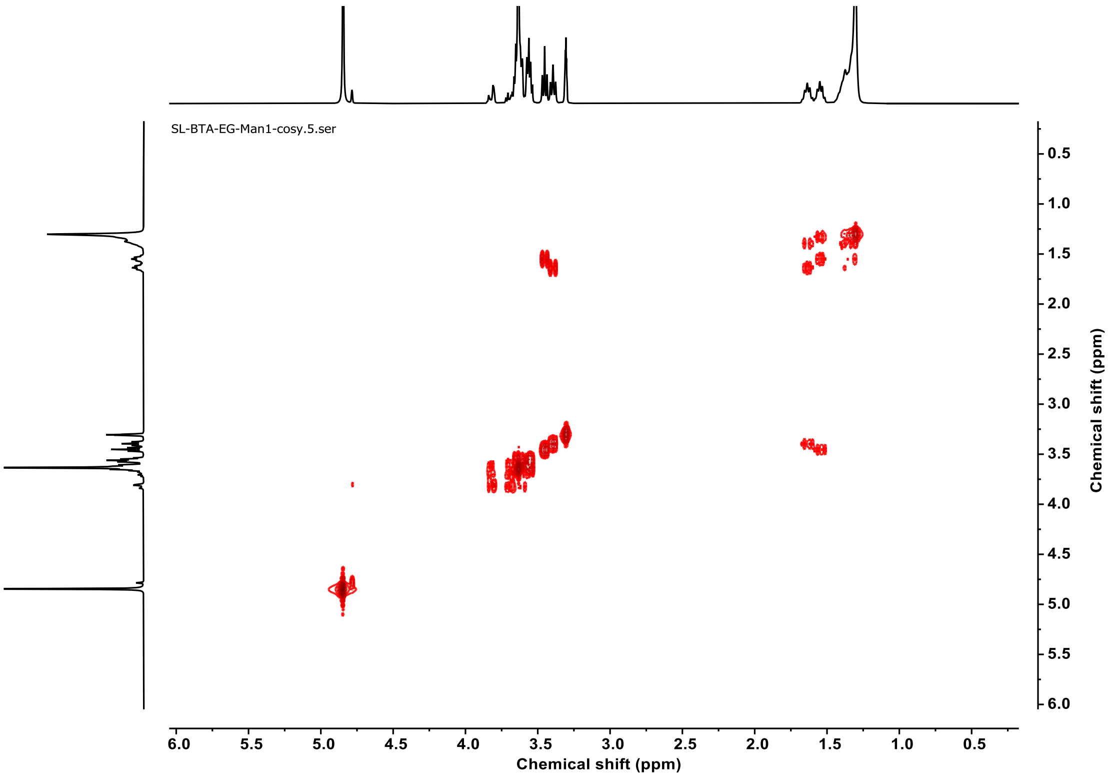
SL-BTA-EG-Man1-cosy.5.ser
0.5 1.0 1.5 2.0 2.5 3.0 3.5 4.0 4.5 5.0 5.5 6.0
Chemical shift (ppm)
6.0 5.5 5.0 4.5 4.0 3.5 3.0 2.5 2.0 1.5 1.0 0.5
Chemical shift (ppm)

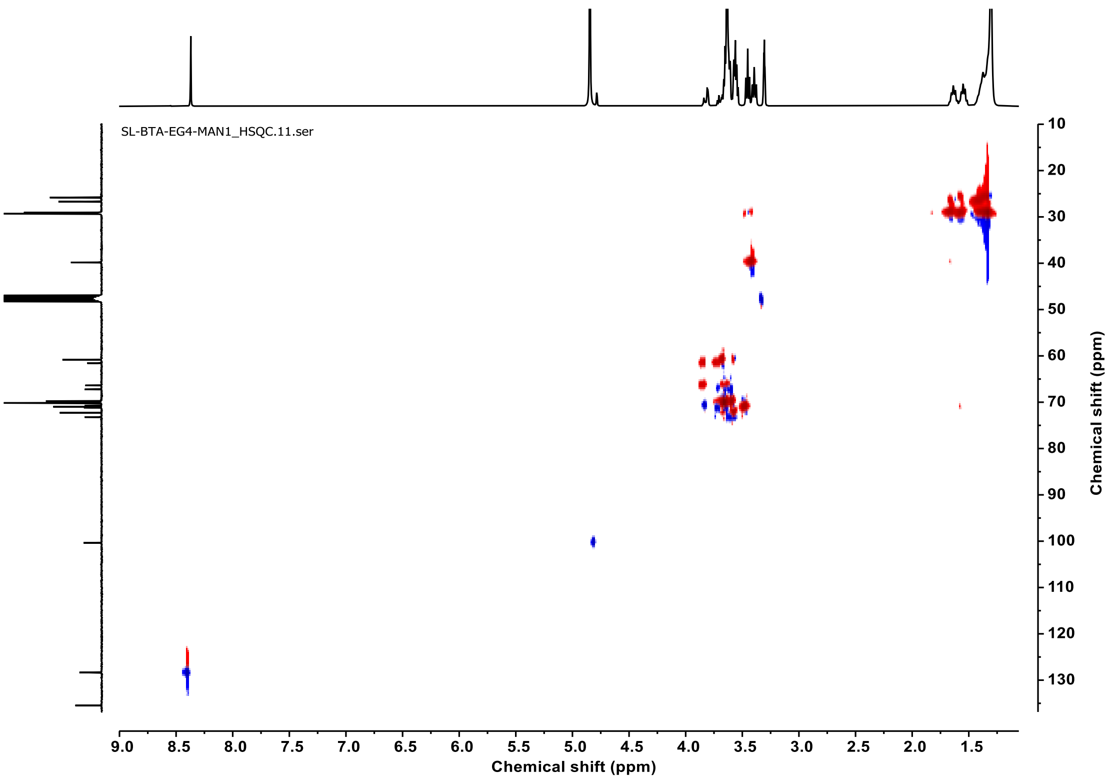

Fig. S1: ^{1}H NMR (400 MHz), ^{13}C NMR (100 MHz), COSY, and HSQC of **M1 BTA** in CD_3OD.

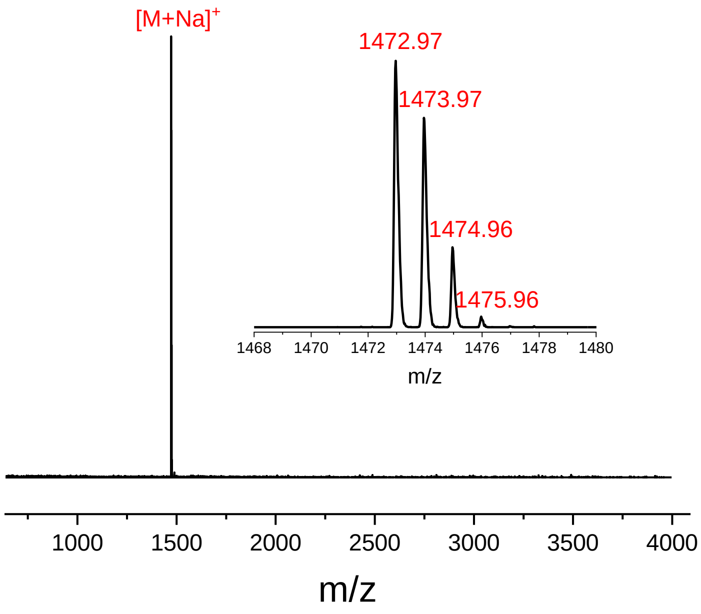

Fig. S2: MALDI-TOF of **M1 BTA** with a zoomed in inset.

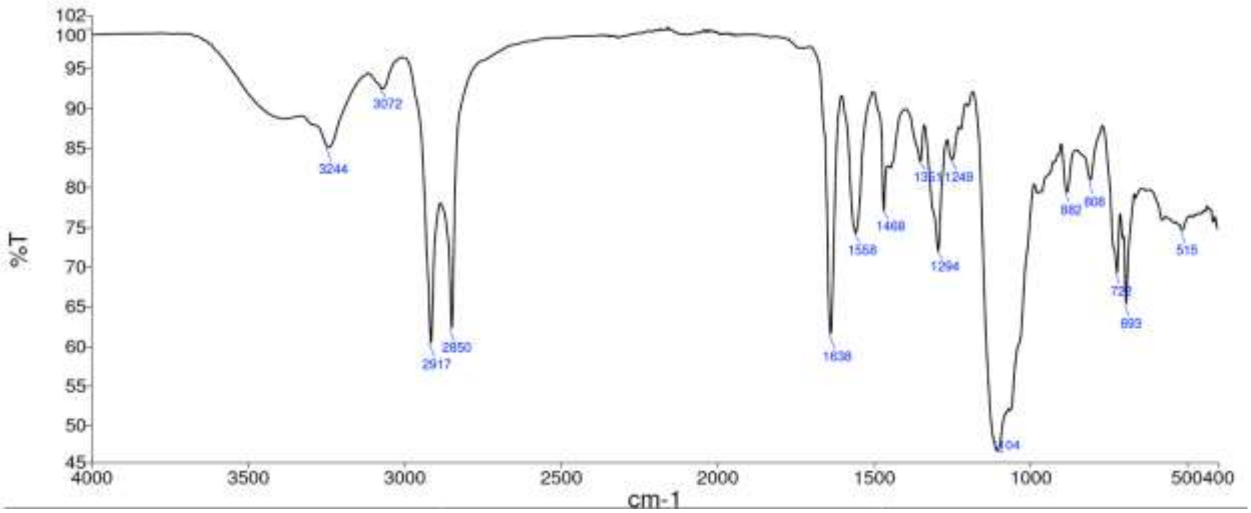

Fig. S3: FT-IR spectrum of **M1 BTA** measured in solid state at room temperature.

M2 BTA

^{1}H NMR (400 MHz, MeOD) δ 8.38 (s, 3H), 4.79 (d, *J* = 1.7 Hz, 2H), 3.87 – 3.83 (m, 2H), 3.81 (d, *J* = 2.8 Hz, 4H), 3.73 – 3.60 (m, 44H), 3.57 (qd, *J* = 5.9, 4.1 Hz, 12H), 3.43 (dt, *J* = 23.6, 6.9 Hz, 12H), 1.60 (dp, *J* = 33.9, 6.8 Hz, 13H), 1.47 – 1.27 (m, 49H).

^{13}C NMR (100 MHz, MeOD) δ 168.59, 136.85, 129.75, 101.75, 74.61, 73.67, 72.57, 72.38, 72.11, 71.62, 71.58, 71.55, 71.53, 71.39, 71.15, 68.63, 67.77, 62.96, 62.22, 41.23, 30.72, 30.69, 30.68, 30.58, 30.46, 28.09, 27.21.

MALDI-TOF-MS: Calculated for $C_{81}H_{149}O_{28}N_3$: 1612.03 g/mol; found $[M+Na]^+$: 1635.04 g/mol.

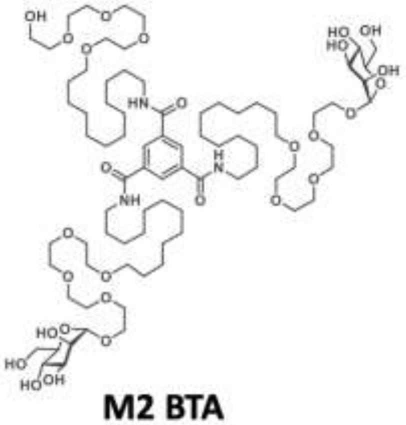

M2 BTA

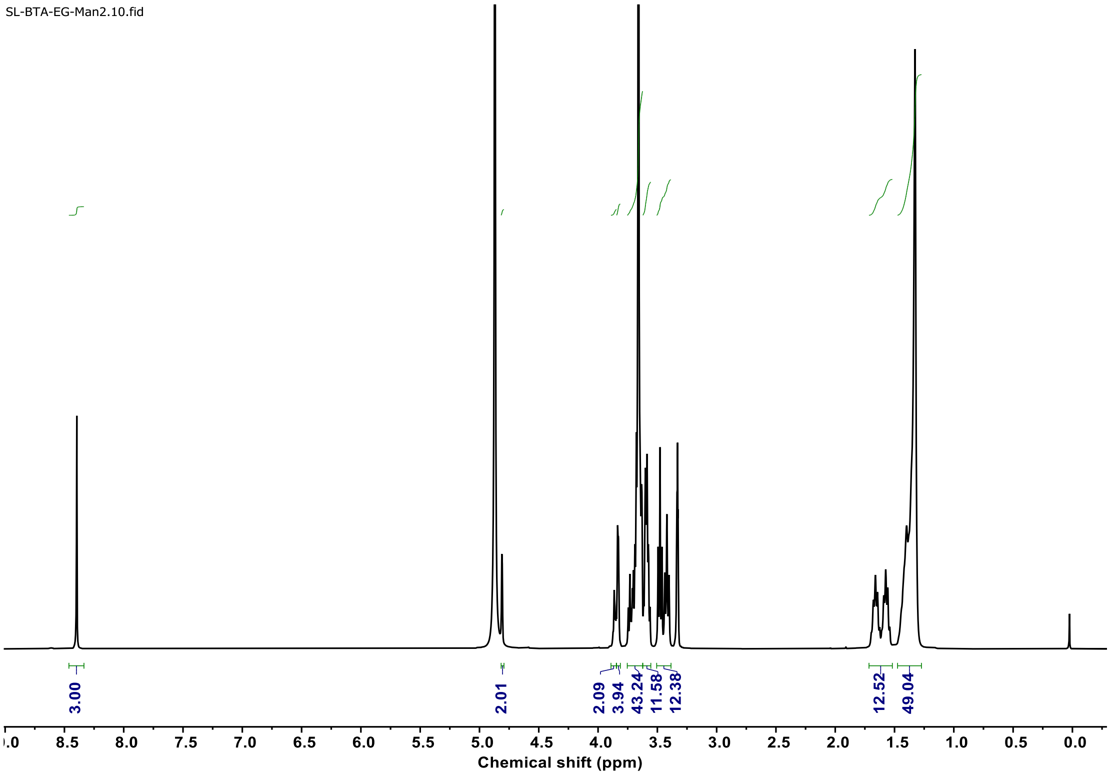

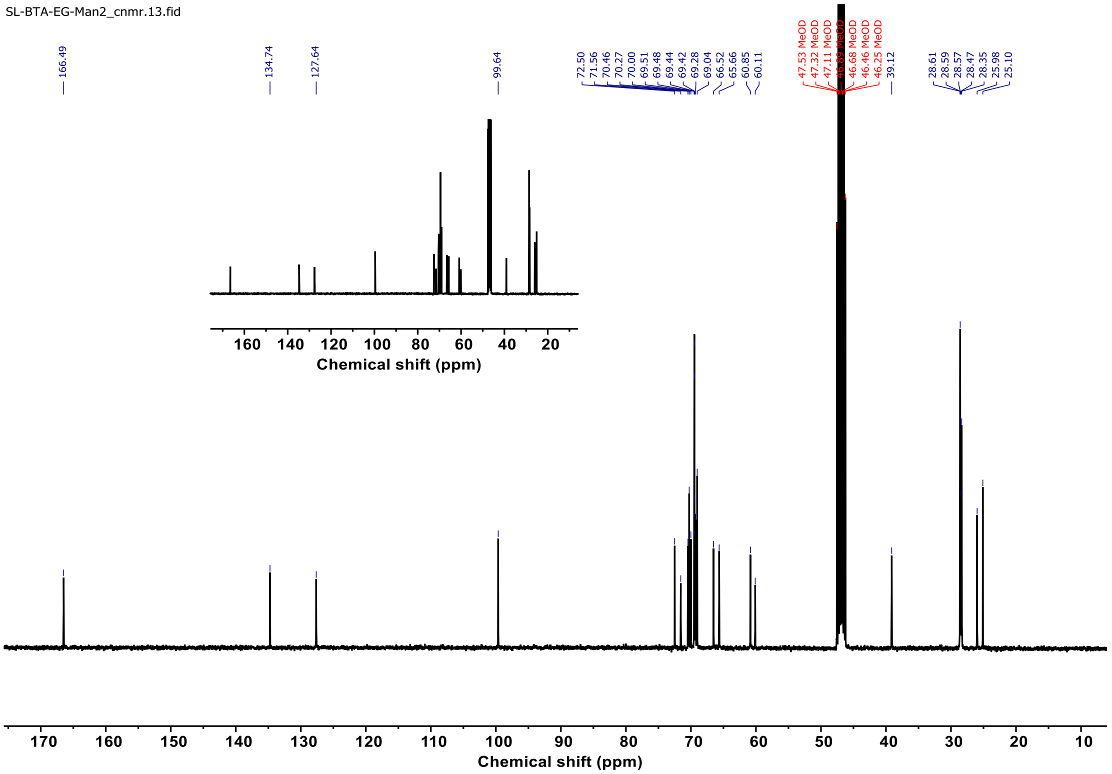

SL-BTA-EG-Man2_cnmr.13.fid
166.49
134.74
127.64
99.64
72.50
71.56
70.46
70.27
70.00
69.51
69.48
69.44
69.42
69.28
69.04
66.52
65.66
60.85
60.11
47.53 MeOD
47.32 MeOD
47.11 MeOD
46.68 MeOD
46.46 MeOD
46.25 MeOD
39.12
28.61
28.59
28.57
28.47
28.35
25.98
25.10
Chemical shift (ppm)
Chemical shift (ppm)

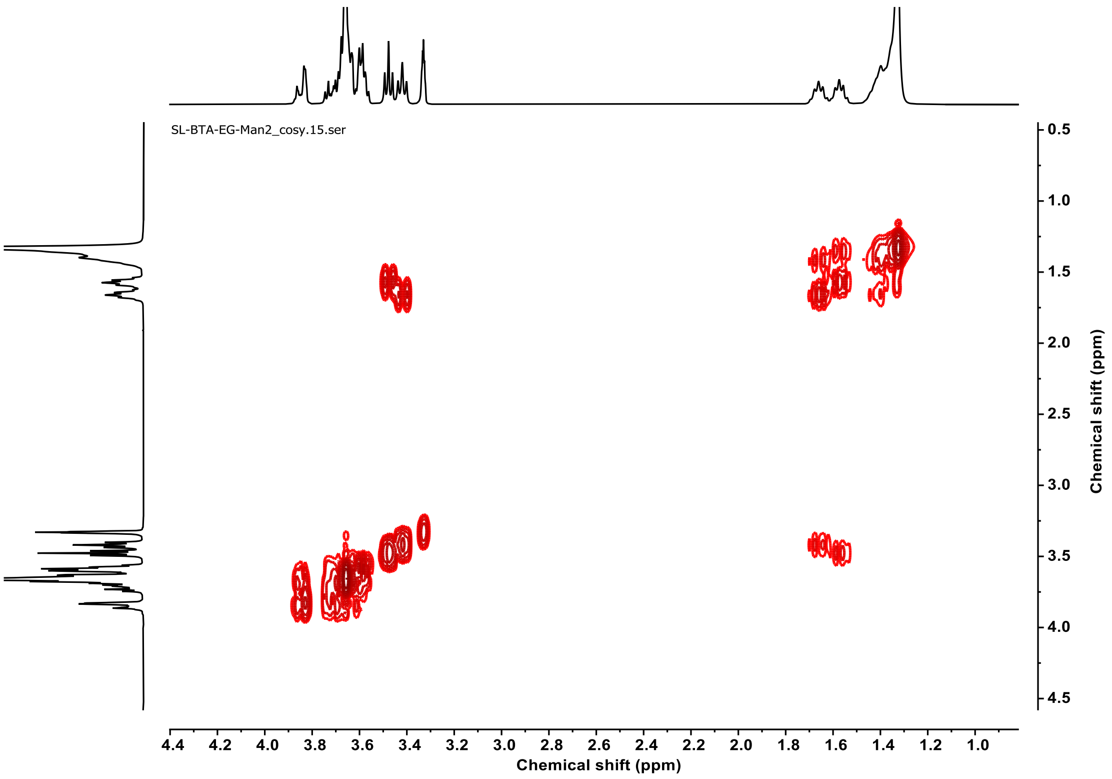

SL-BTA-EG-Man2_cosy.15.ser
Chemical shift (ppm)
Chemical shift (ppm)

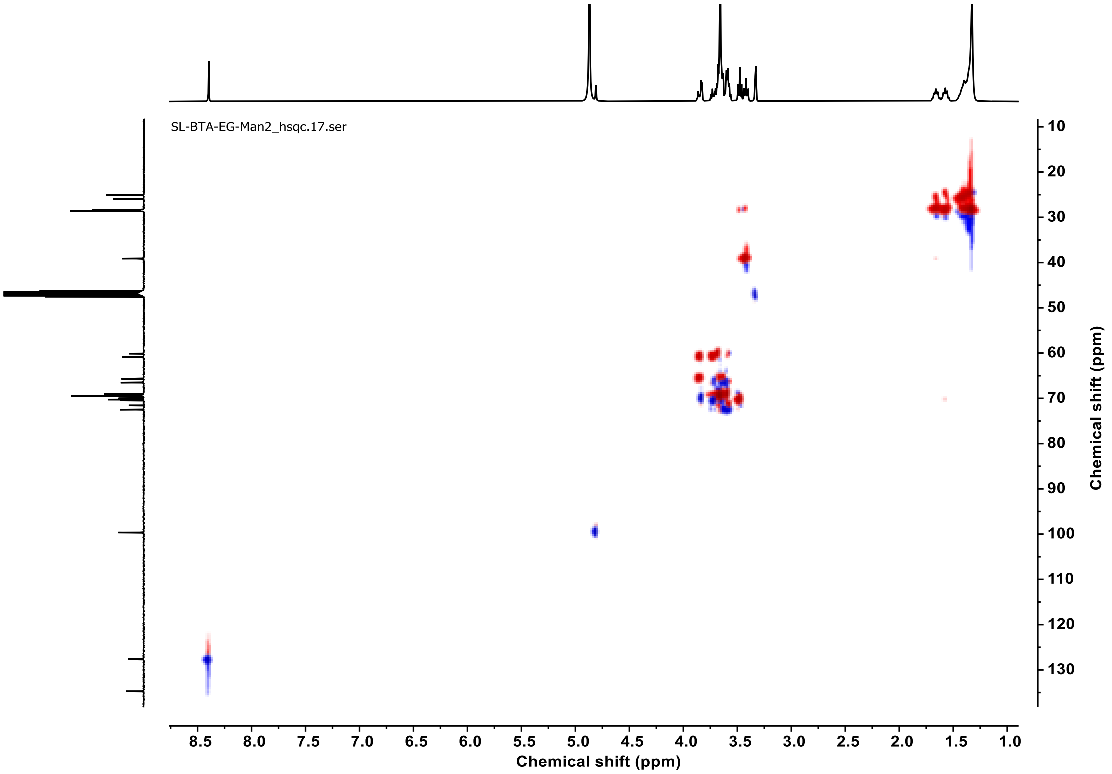

Fig. S4: ^{1}H NMR (400 MHz), ^{13}C NMR (100 MHz), COSY, and HSQC of **M2 BTA** in CD_3OD.

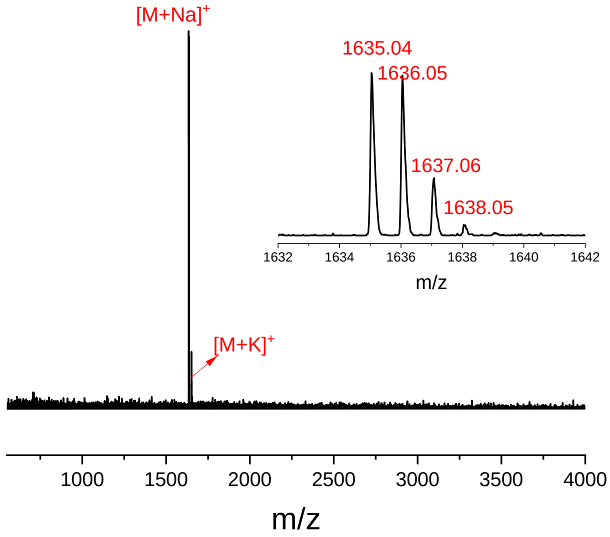

Fig. S5: MALDI-TOF of **M2 BTA** with a zoomed in inset.

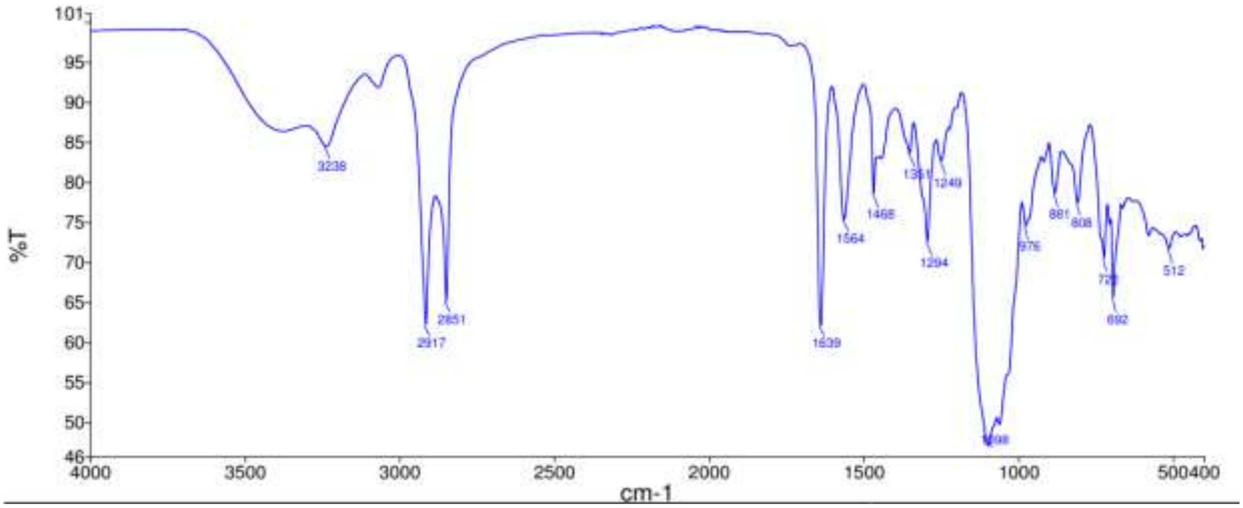

Fig. S6: FT-IR spectrum of **M2 BTA** measured in solid state at room temperature.

Sample preparation

A) BTA homo-assemblies: The desired amount of solid BTA material was weighed into a clean glass vial equipped with stirring bar and dissolved in MQ water to yield the predetermined concentration. The mixture was stirred at 80 °C for 15 min. The resulting hot mixture was vortexed for 15 seconds and allowed to equilibrate at room temperature overnight. The next day, characterization or cell experiments were performed. Special handling was however required for **M1 BTA**, which was also sonicated and vortexed for 30 seconds several times during the heating procedure.

B) BTA co-assemblies: Directly after heating of the homo-assemblies, adequate amounts of **nBTA** and the desired mannose-decorated BTA were pipetted together. To ensure homogeneous mixing, the resulting solution was pipetted up and down several times and heated while stirring at 80 °C for another 15 min. The resulting mixture was vortexed for 15 seconds after heating and allowed to equilibrate at room temperature overnight. The next day, characterization or cell experiments were performed.

C) BTA hydrogel: The desired amount of solid mannose BTA material was weighed into a clean glass vial equipped with stirring bar and dissolved in adequate amount of MQ water to yield the final gel concentration. The mixture was stirred at 80 °C for 15-30 min. Special handling was executed for **M1 BTA**, which was also sonicated and vortexed for 30 seconds several times during the heating procedure. After heating, the resulting mixture was vortexed for 15 seconds and left for equilibration at 4 °C overnight. The next day, rheological experiments were performed.

D) BTA-Cy3 or **BTA-Cy5 containing solution:** BTA solution without dye was prepared as described above (method A). Appropriate volume of **BTA-Cy3** or **BTA-Cy5** stock solution (1 mM in MeOH) was added to the hot mixture, followed by the equilibration at 45 °C with stirring for 15 min. The mixture was subsequently equilibrated in the dark at room temperature overnight. The next day, characterization or cell experiments were performed. Heating with dye-containing solutions at 80 °C was avoided as molecules might degrade.

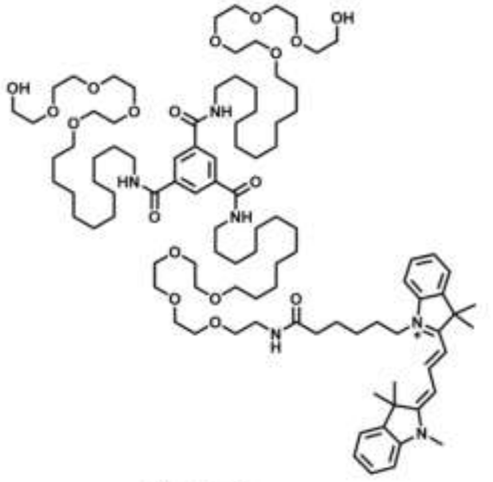

BTA-Cy3

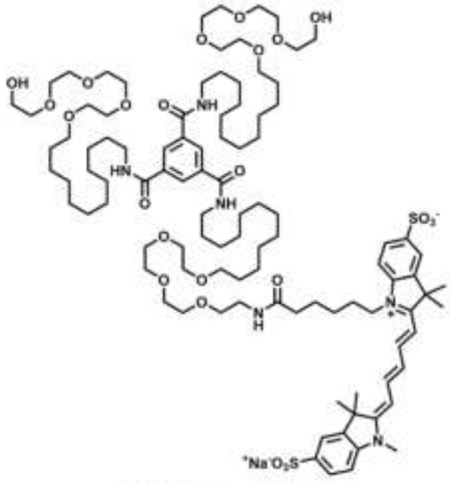

BTA-Cy5 sulfo

Supplementary figures

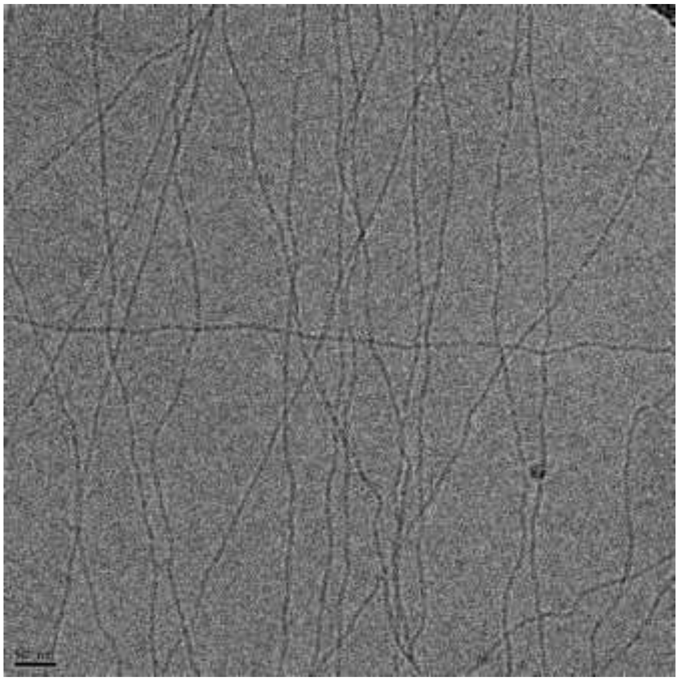

Fig. S7. Cryo-TEM image of **M1 BTA** (500 µM), show micrometer long fibers. The black spherical particles are ice-crystals. Scale bar = 50 nm.

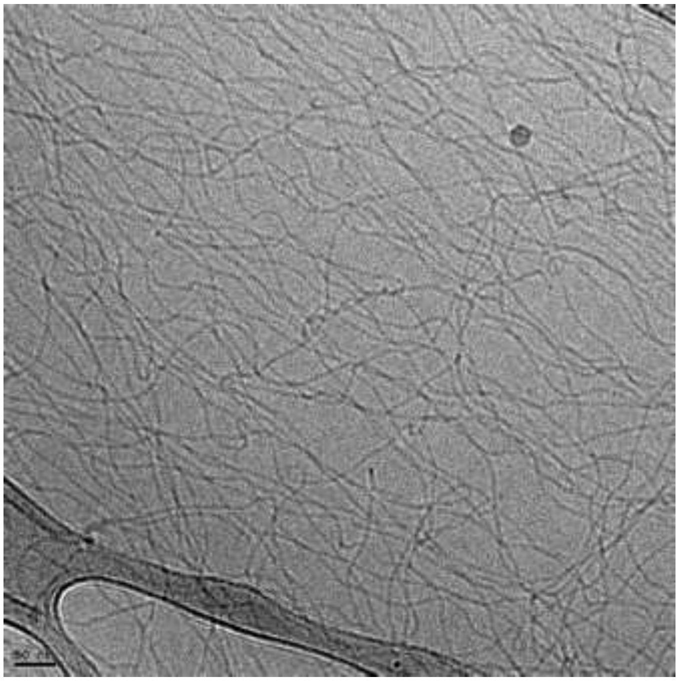

Fig. S8. Cryo-TEM image of **M2 BTA** (500 µM), show micrometer long fibers. The black spherical particles are ice-crystals. Scale bar = 50 nm.

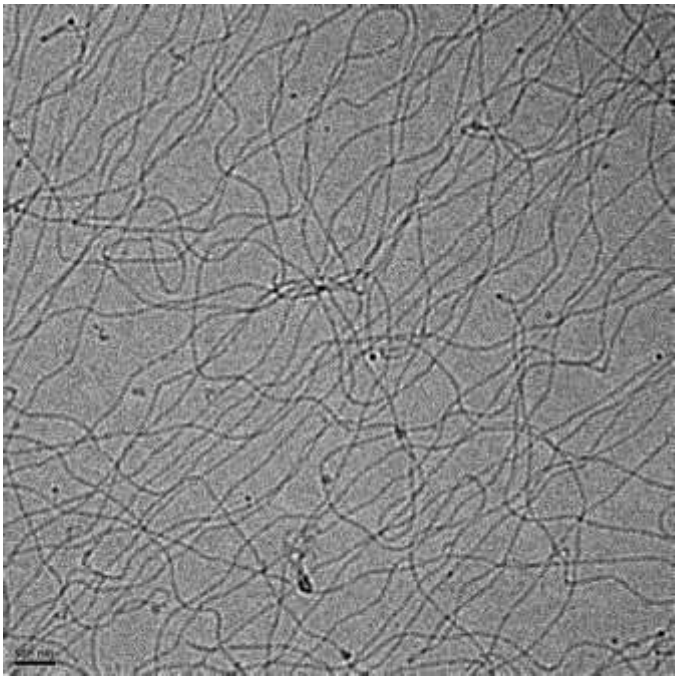

Fig. S9. Cryo-TEM image of **Copolymer_1** with **nBTA**:**M3 BTA = 2:1** (BTA = 500 µM), show micrometer long fibers. The black spherical particles are ice-crystals. Scale bar = 50 nm.

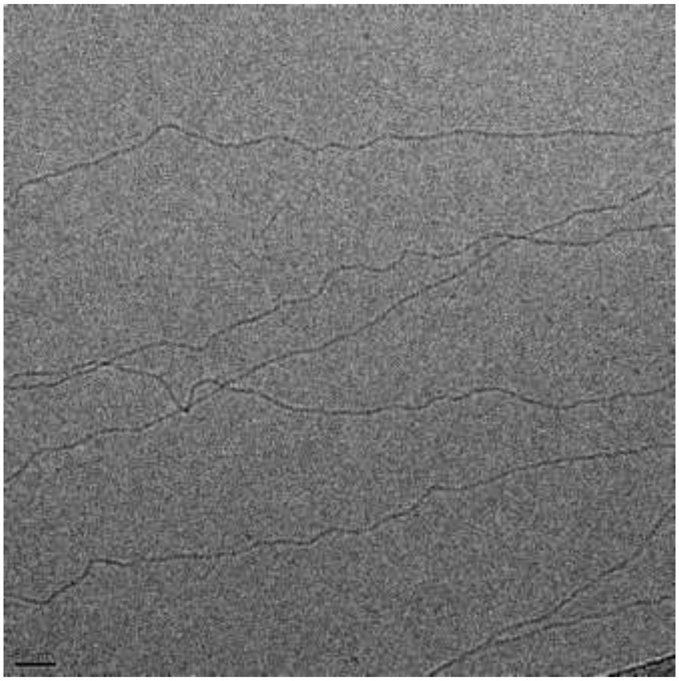

Fig. S10. Cryo-TEM image of **Copolymer_2** with **nBTA**:**M3 BTA = 1:2** (BTA = 500 µM), show micrometer long fibers. Scale bar = 50 nm.

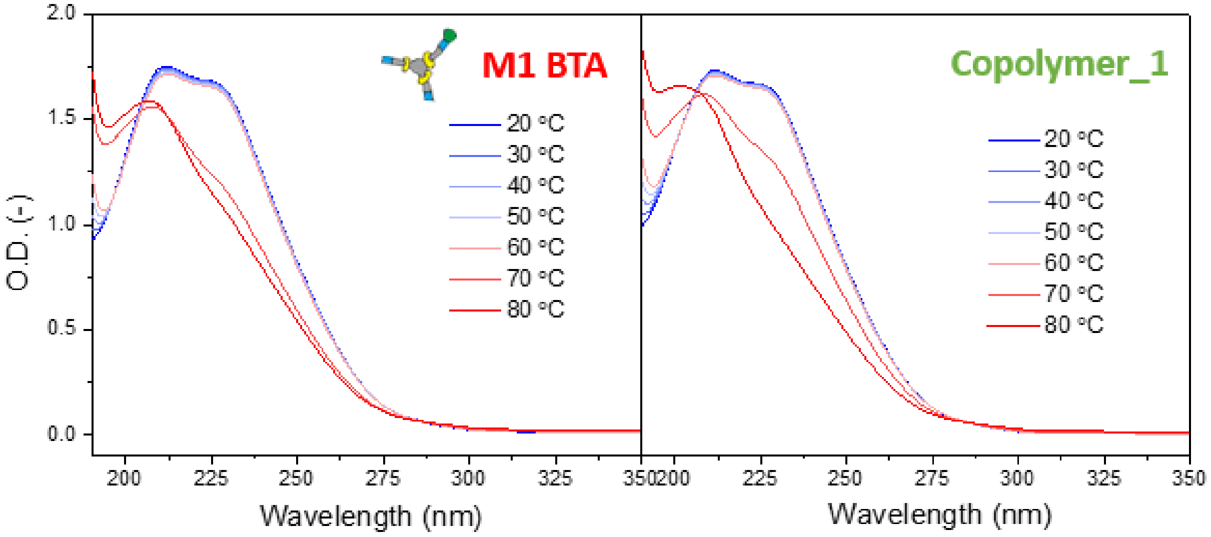

Fig. S11. Variable temperature UV spectra (50 µM) of **M1 BTA (left)** and **Copolymer_1 (right)** in MQ-H_2O.

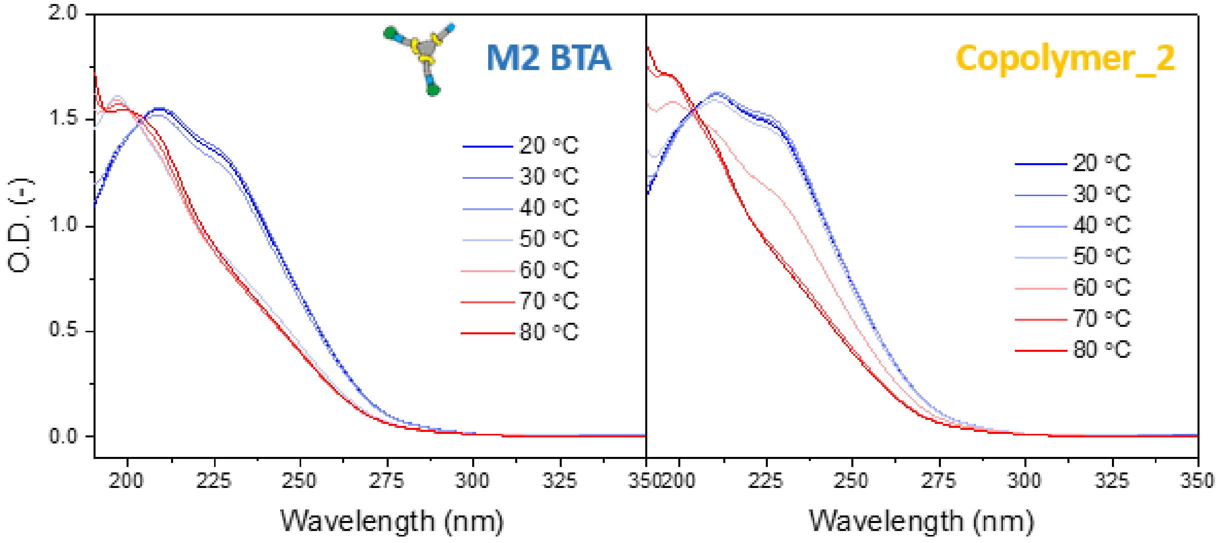

Fig. S12. Variable temperature UV spectra (50 µM) of **M2 BTA (left)** and **Copolymer_2 (right)** in MQ-H_2O.

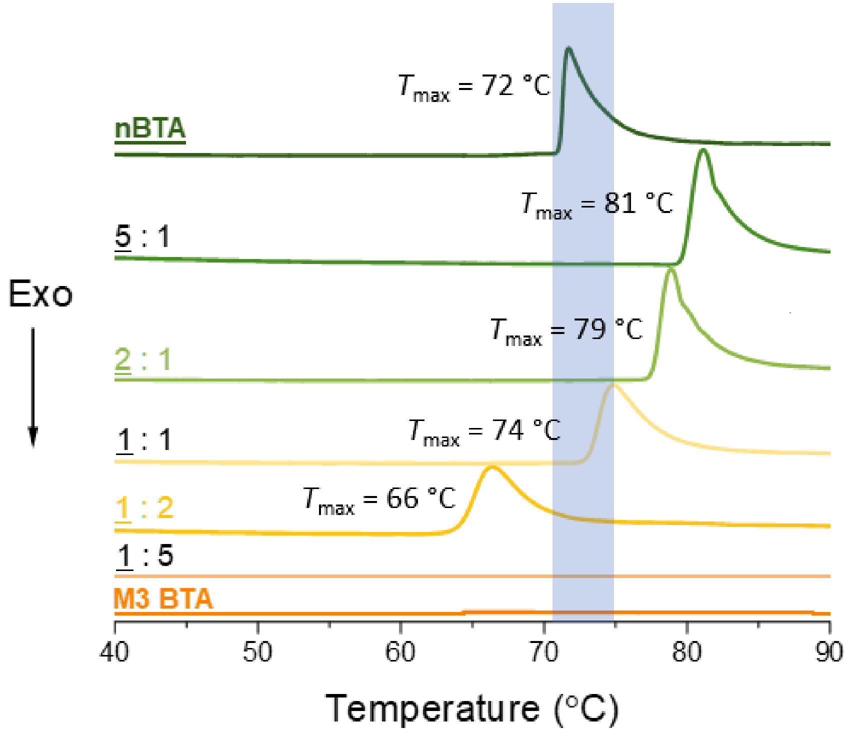

Fig. S13. MicroDSC profiles (500 µM) of **nBTA**, **M3 BTA** and their copolymers in MQ-H_2O revealing the endothermic peaks (scanning rate = 60 °C/h).

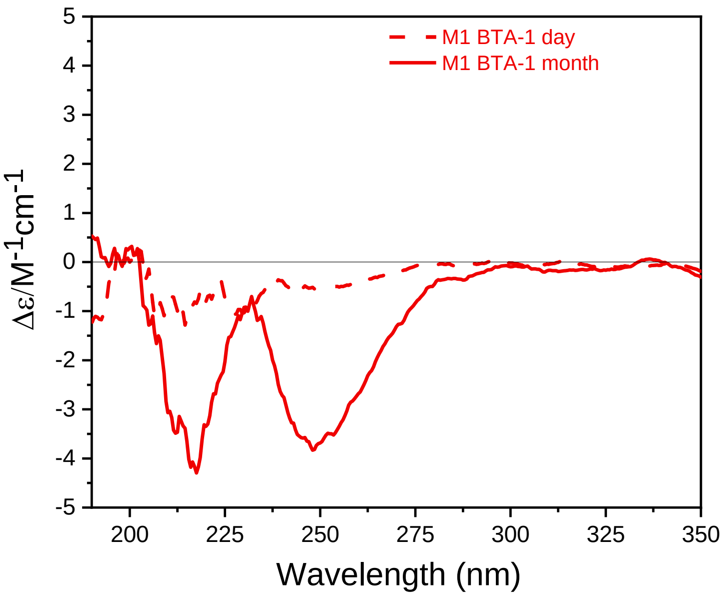

Fig. S14. CD spectrum (50 µM) of **M1 BTA** in MQ-H_2O after ageing.

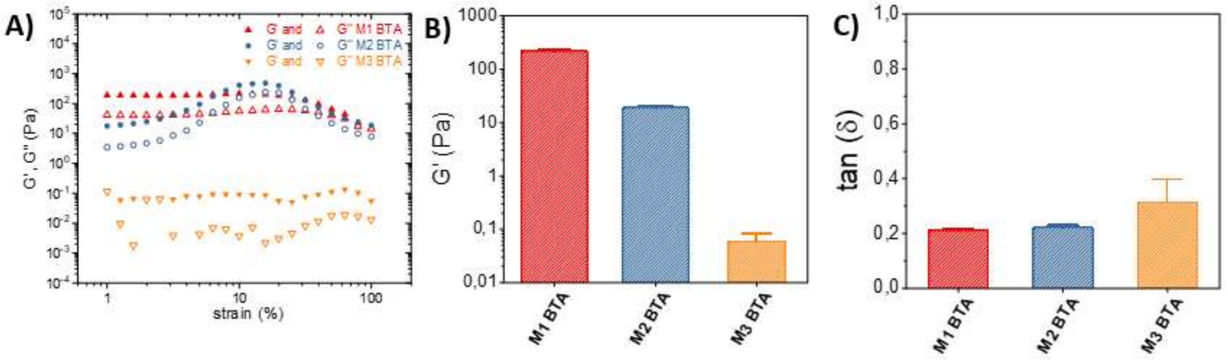

Fig. S15. Mechanical characterization of supramolecular mannose BTA hydrogels measured at 5 w/v% at room temperature using rheology, with in **A)** strain sweep plot at 1% strain showing the storage (G') and loss modulus (G"), in **B)** G' quantified and in **C)** the loss factor (tan(δ) showing the ratio of viscous to elastic response, both obtained at 1 rad/s and 1% strain.

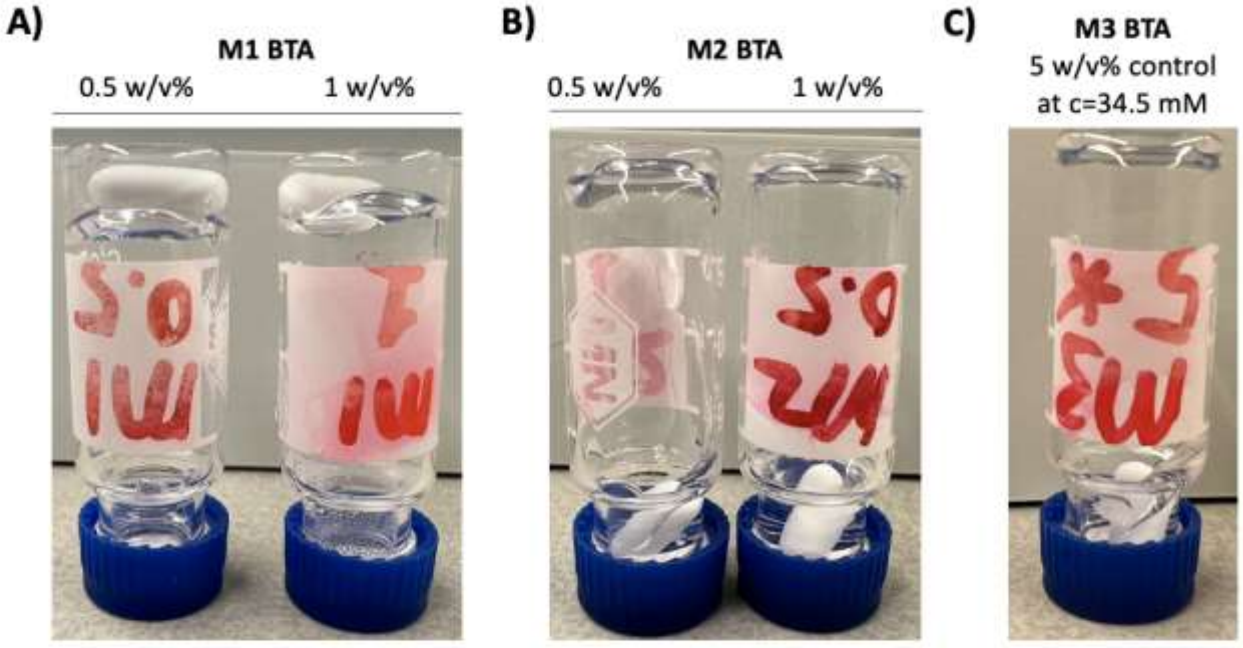

Fig. S16. Vial inversion tests of **A)** 0.5 and 1 w/v% **M1 BTA** samples, revealing gels that can hold their own weight, **B)** 0.5 and 1 w/v% **M2 BTA** samples, which are liquids that cannot hold their own weight and **C)** the 5 w/v% **M3 BTA** control sample, containing equal molar concentration as **M1 BTA (34.5 mM)** at 5 w/v% concentration, showing it is still a liquid and cannot withstand its own weight.

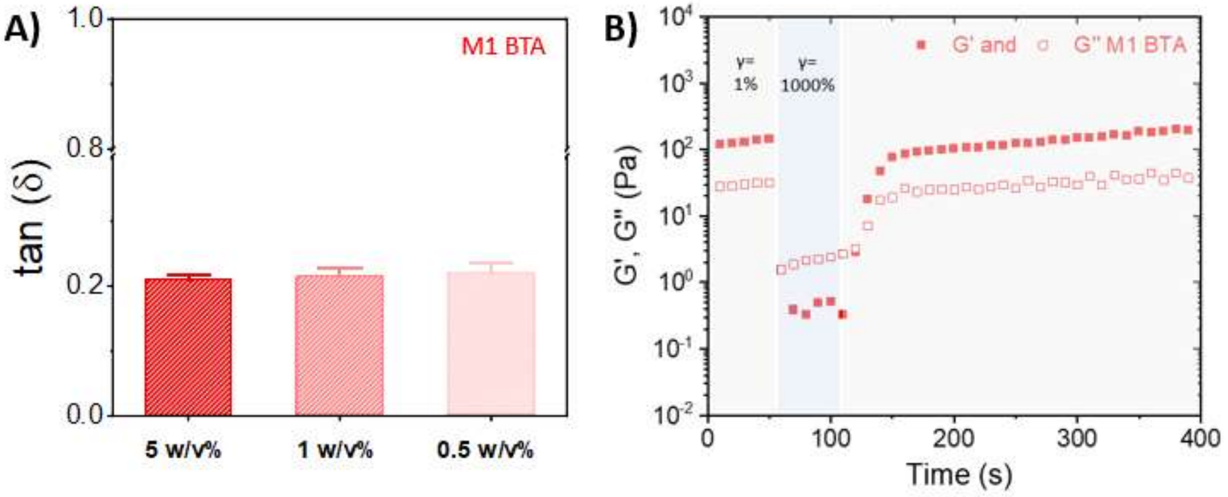

Fig. S17. Mechanical characterization and self-healing behavior of supramolecular **M1 BTA** hydrogel. **A**) The loss factor (tan(δ)), i.e. the ratio of viscous to elastic response, measured at different concentrations. **B)** Self-healing rheological experiment of **M1 BTA** hydrogel. The gel is first subjected to 1% strain, after which the strain is increased to 1000% to completely break the gel. After removing the extreme strain, the gel self-heals to its original stiffness. 1 rad/s is applied during the whole cycle.

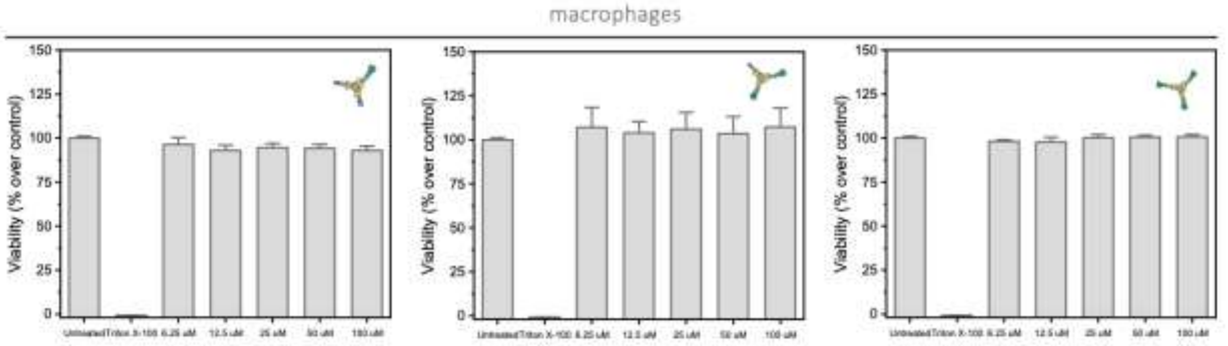

Fig. S18. Toxicity test of different concentrations of mannose BTA assemblies towards RAW 264.7 macrophages as determined with a resazurin assay (50,000 RAW264.7 macrophages/well in 96-well plate). The BTA assemblies were exposed to the cells in complete culture medium for 24 h. The cell viability is normalized on cells that were maintained in untreated medium (i.e. medium control). Exposure of the cells to 0.5 v/v% Triton X-100 in PBS was used as a negative control.

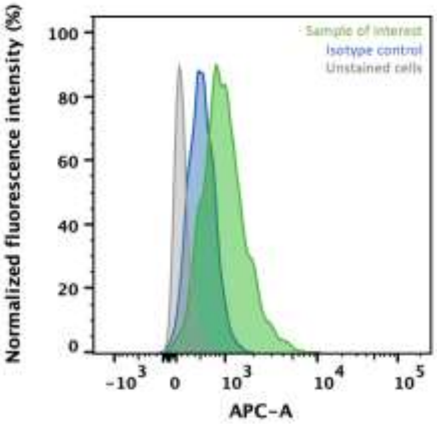

Fig. S19. Flow cytometry experiment to investigate the presence of the CD206 on RAW macrophages. Visualized in green, the sample of interest containing the specific anti-CD206 antibody, in blue the isotype control and in grey the unstained control, revealing that 50% of RAW 264.7 macrophages is CD206+.

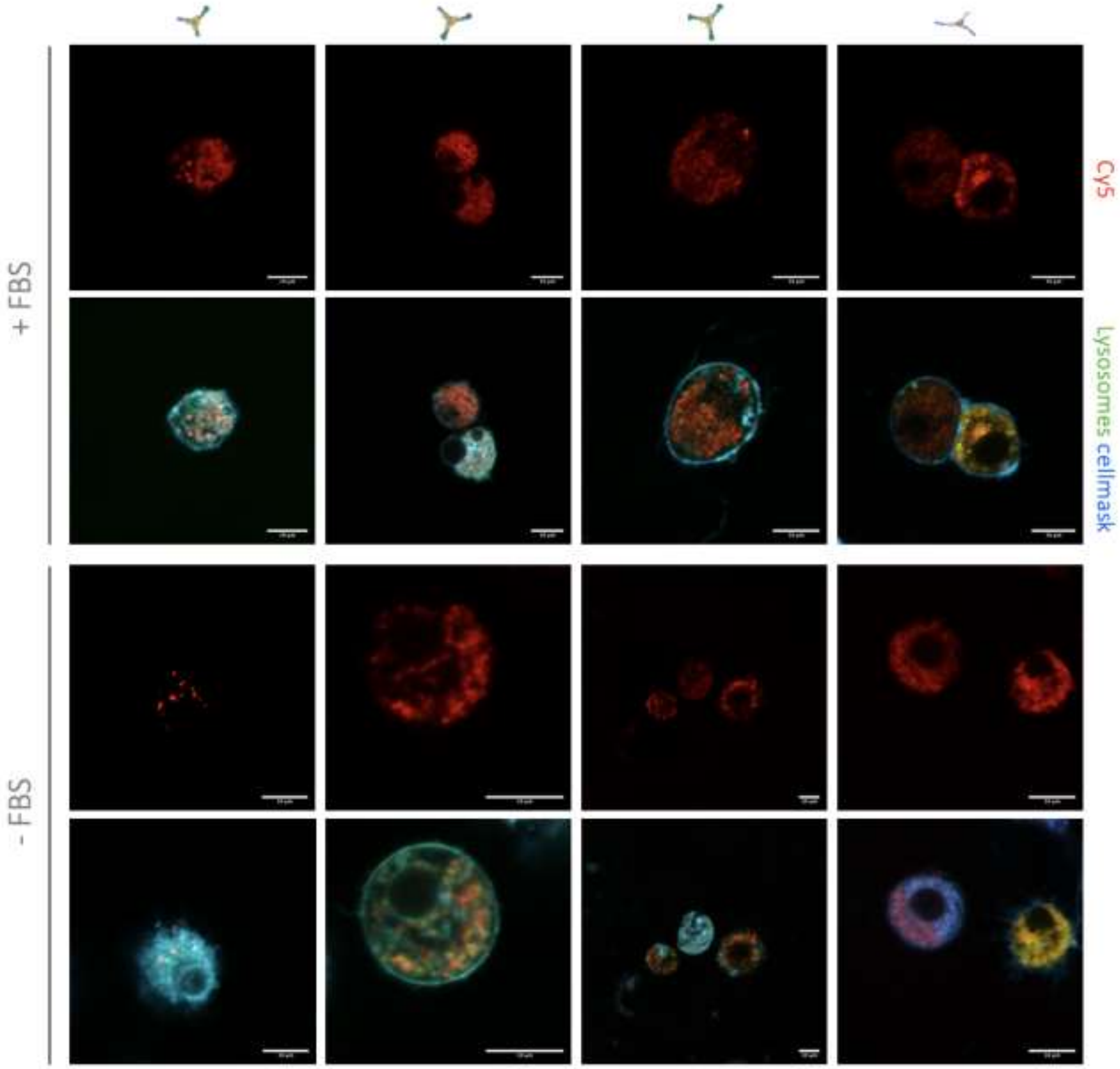

Fig. S20. Internalization experiment in which the RAW 264.7 macrophages were incubated for 2h with Cy5-labeled mannose BTAs (c=25 μM total, with 5% **BTA-Cy5**) in presence and absence of BSA-containing FBS, and stained thereafter with a lysosomal and cell mask in green and blue, respectively. Scale bar = 10 μm.

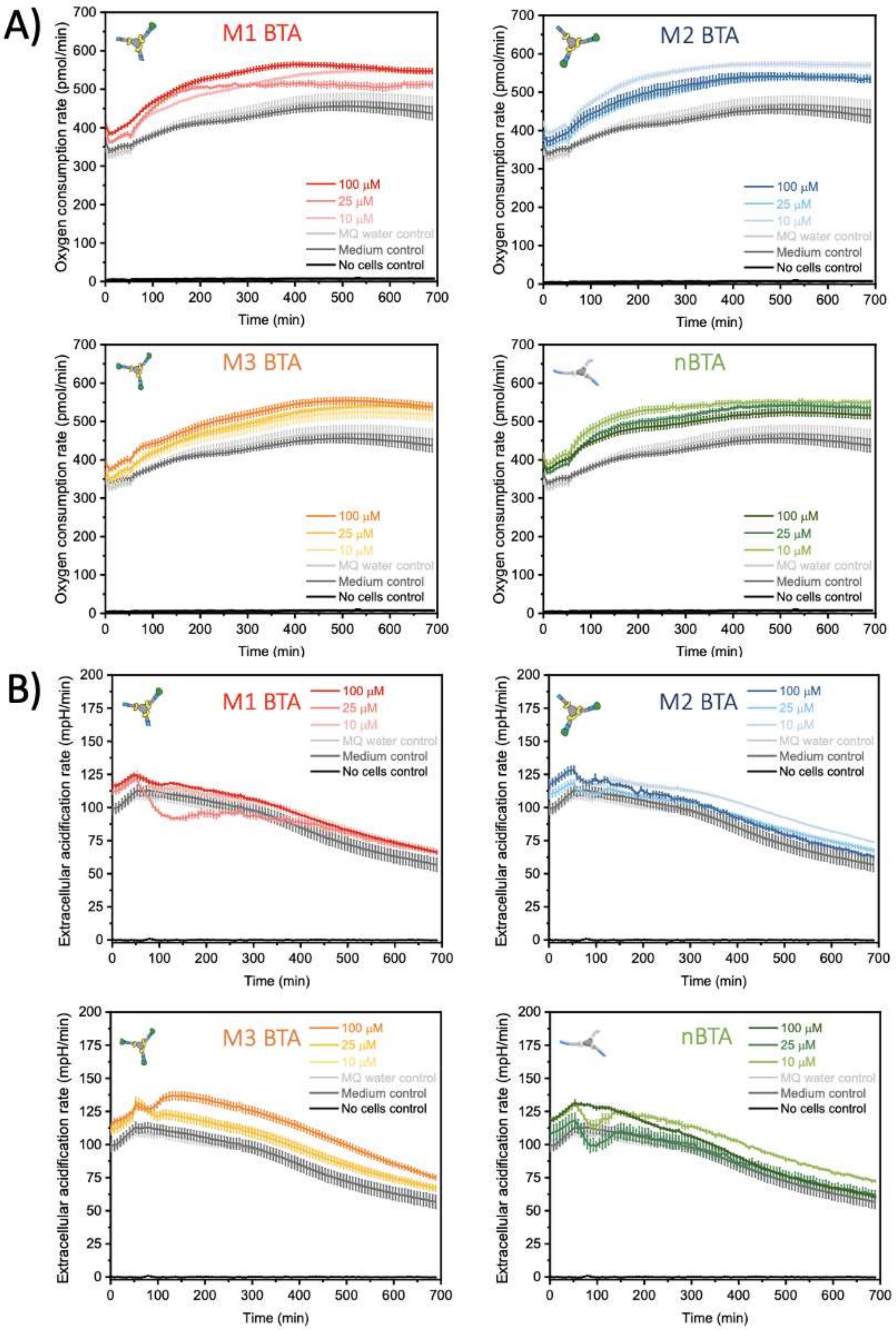

Fig. S21. Seahorse metabolism flux assay to study the metabolic changes of RAW 264.7 macrophages (6 x 10^5 cells/well) during a 10 h incubation period with the mannose BTA assemblies, monitored using the Seahorse XFe96 Analyzer (Agilent), showing **A)** the oxygen consumption rate (OCR) (pmol/min) and **B)** the extracellular acidification rate (ECAR) (mpH/min).

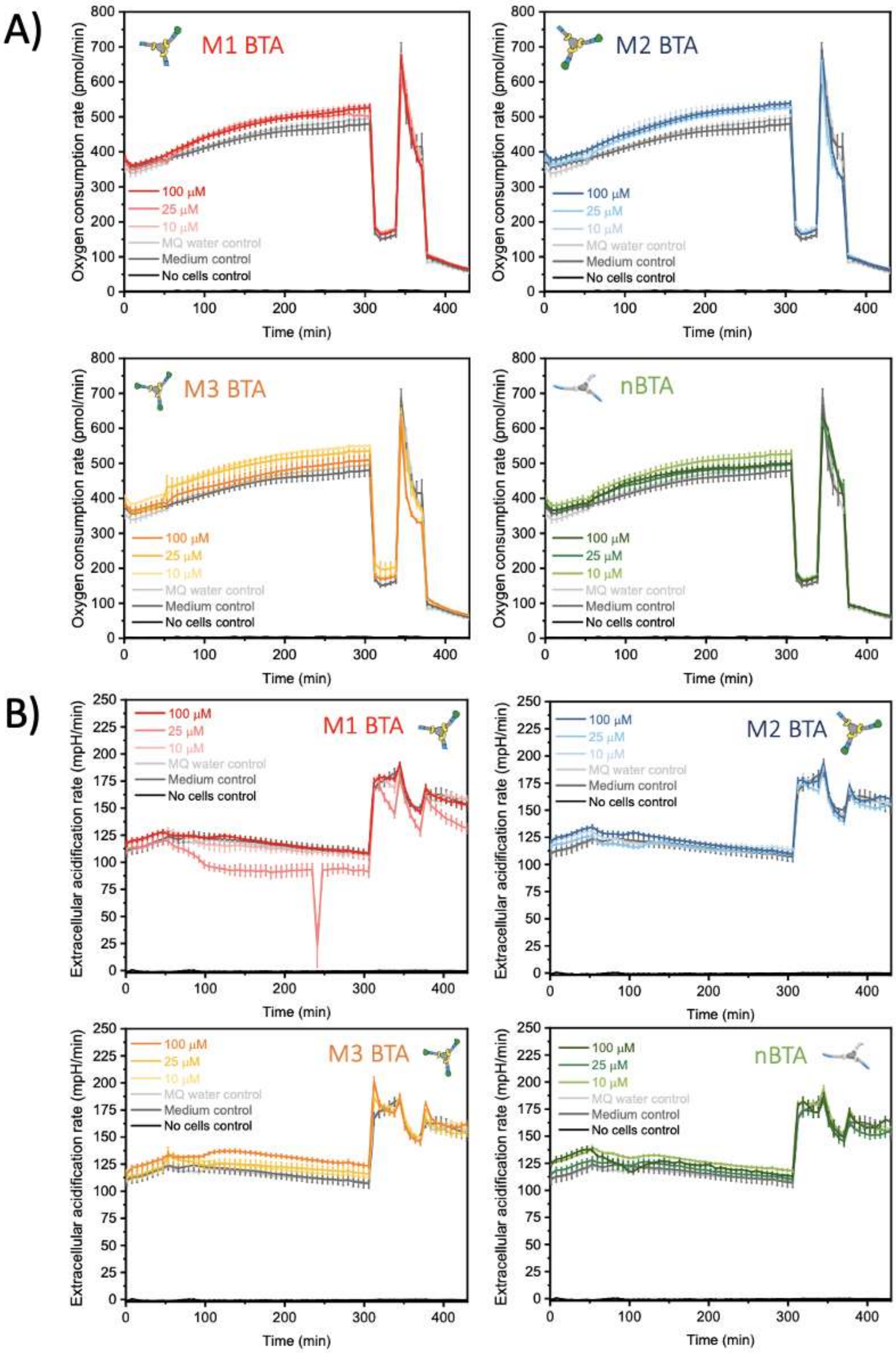

Fig. S22. Seahorse XF Cell Mito Stress Assay to investigate the mitochondrial function of RAW 264.7 macrophages (6 x 10^5 cells/well) treated with mannose BTAs for 4 h, showing **A)** the OCR (pmol/min) and **B)** the ECAR (mpH/min).

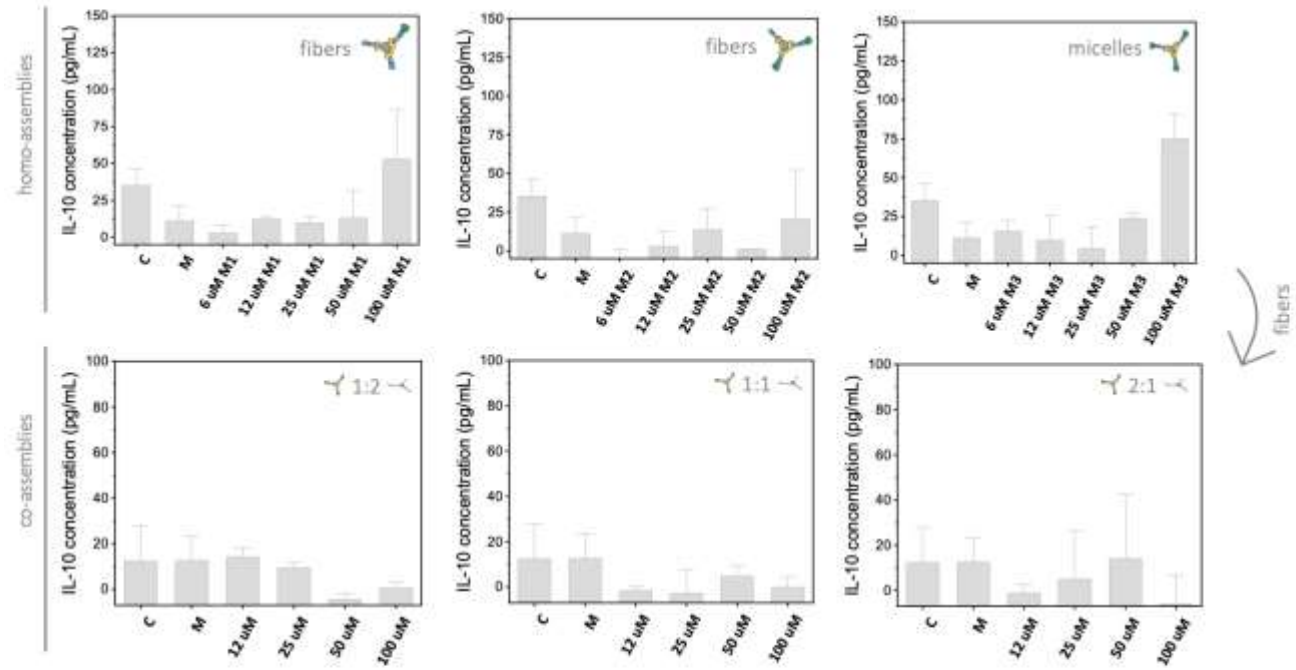

Fig. S23. Representative graphs of ELISA assay to quantify the release of IL-10 in RAW 264.7 macrophages after treatment with different concentrations of homo-assemblies (top) and their co-assemblies (bottom) (50,000 RAW 264.7 macrophages/well in 96-well plate). The BTA assemblies were exposed to the cells in complete culture medium for 24 h. Untreated cells and wells containing only medium were both used as negative controls.

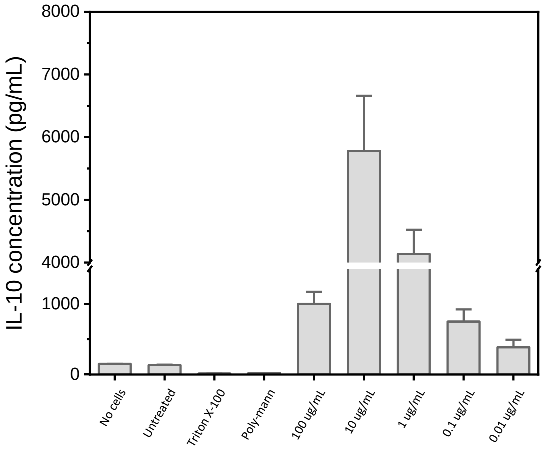

Fig. S24. ELISA assay to quantify the release of IL-10 in RAW 264.7 macrophages after treatment with mannan (poly-mannose) and different LPS concentrations as controls (50,000 RAW 264.7 macrophages/well in 96-well plate). The BTA assemblies were exposed to the cells in complete culture medium for 24 h. Untreated cells and wells containing only medium were both used as negative controls.

References

1 S. B. Pruett, R. Fan, Q. Zheng and C. Schwab, *Alcohol*, 2006, **37**, 1–8.

2 L. Albertazzi, D. van der Zwaag, C. M. A. Leenders, R. Fitzner, R. W. van der Hofstad and E. W. Meijer, *Science,* 2014, **344**, 491–495.

3 L. Su, C. Wang, F. Polzer, Y. Lu, G. Chen and M. Jiang, *ACS Macro Lett*, 2014, **3**, 534–539.

4 C. M. A. Leenders, L. Albertazzi, T. Mes, M. M. E. Koenigs, A. R. A. Palmans and E. W. Meijer, *Chemical Communications*, 2013, **49**, 1963–1965.

5 S. I. S. Hendrikse, L. Su, T. P. Hogervorst, R. P. M. Lafleur, X. Lou, G. A. Van Der Marel, J. D. C. Codee and E. W. Meijer, *J Am Chem Soc*, 2019, **141**, 13877–13886.
